# Neuronal fatty acid oxidation fuels memory after intensive learning in *Drosophila*

**DOI:** 10.1038/s42255-025-01416-5

**Published:** 2025-12-10

**Authors:** Alice Pavlowsky, Bryon Silva, Ruchira Basu, Amandine Correia Delecourt, David Geny, Lydia Danglot, Pierre-Yves Plaçais, Thomas Preat

**Affiliations:** 1 Energy & Memory, Brain Plasticity Unit, CNRS, ESPCI Paris, PSL Research University, Paris, France; 2 NeurImag Core Facility, Institute of Psychiatry and Neuroscience of Paris, INSERM U1266, Université Paris Cité, Paris, France

**Keywords:** Classical conditioning, Energy metabolism, Metabolism, Learning and memory, Mitochondria

## Abstract

Metabolic flexibility allows cells to adapt to different fuel sources, which is particularly important for cells with high metabolic demands^1–3^. In contrast, neurons, which are major energy consumers, are considered to rely essentially on glucose and its derivatives to support their metabolism. Here, using *Drosophila melanogaster*, we show that memory formed after intensive massed training is dependent on mitochondrial fatty acid (FA) β-oxidation to produce ATP in neurons of the mushroom body (MB), a major integrative centre in insect brains. We identify cortex glia as the provider of lipids to sustain the usage of FAs for this type of memory. Furthermore, we demonstrate that massed training is associated with mitochondria network remodelling in the soma of MB neurons, resulting in increased mitochondrial size. Artificially increasing mitochondria size in adult MB neurons increases ATP production in their soma and, at the behavioural level, strikingly results in improved memory performance after massed training. These findings challenge the prevailing view that neurons are unable to use FAs for energy production, revealing, on the contrary, that in vivo neuronal FA oxidation has an essential role in cognitive function, including memory formation.

## Abstract (web summary)

Neurons are shown to use fatty acid β-oxidation as a fuel source for memory formation upon intensive learning in *Drosophila*, challenging the view that neurons are unable to use fatty acids for energy production.

## Main

In mammals, fatty acid (FA) β-oxidation contributes to up to 20% of total brain oxidative metabolism^4^, and glial cells are viewed as the major brain cell type to use FAs for energy production^5–8^. In contrast, neurons, which are major energy consumers, are considered to rely essentially on glucose and its derivatives to support their metabolism^1–3^. Despite the toxicity associated with FA metabolism^6,7^, there is increasing evidence of an FA internal store in neurons^9,10^, and impairing lipolysis from this internal store in vitro impairs neuronal energy production^11,12^. However, whether neuronal mitochondria actually perform β-oxidation in vivo to support cognitive function remains unknown. To address this question, we used classical Pavlovian olfactory conditioning in *Drosophila melanogaster*, involving the paired delivery of an odorant and electric shocks. Repeated consecutive learning sessions (intensive massed training) induce a memory that decays within 1–2 days (historically called anaesthesia-resistant memory (ARM)^13^), while repeated sessions spaced in time (intensive spaced training) lead to a memory that lasts for up to 1 week^13^. This latter type of memory, classically called long-term memory (LTM), is dependent on protein synthesis^13^ and requires an extended post-training upregulation of pyruvate mitochondrial metabolism in mushroom body (MB) neurons^14,15^, the major olfactory memory centre in *Drosophila*^16^. In contrast, we showed that the memory formed after intensive massed training, which resembles cramming-like learning, is dependent neither on mitochondrial pyruvate metabolism^14^ nor on ketone body oxidation^17^. This leaves the possibility that MB neurons would use FAs as an energy substrate in that context.

**Fig. 1:**
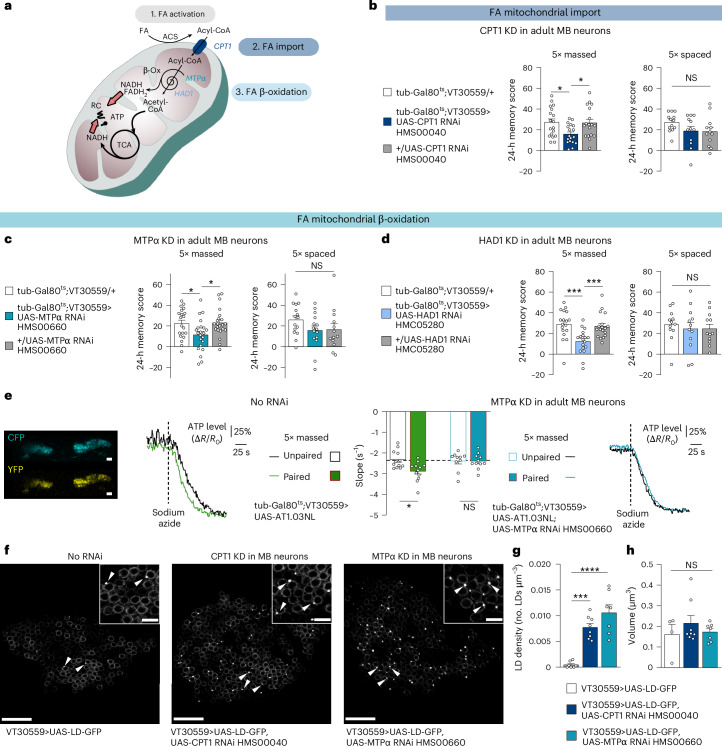
FA mitochondrial β-oxidation is required in MB neurons for memory after massed training. **a**, Before mitochondrial import, FAs are activated by acyl-CoA synthetase (ACS) into acyl-CoA, which is then shuttled into the mitochondrial matrix by the carnitine shuttle system. The outer mitochondrial membrane component of this shuttle, CPT1, catalyses the rate-limiting step of FA import. β-oxidation (β-Ox) is a cyclic process: each cycle shortens the acyl-CoA by two carbons and releases an acetyl-CoA together with reduced cofactors (FADH_2_ and NADH) that directly feed into (red arrows) the respiratory chain (RC) to produce ATP. The β-oxidation machinery harbours different chain length-specific enzymes organized into different functional complexes: MTPα is part of the MTP, an enzymatic complex attached to the inner mitochondrial membrane that shortens long-chain FAs to medium-chain lengths; they are then oxidized by a matrix system containing soluble enzymes for medium- and short-chain FAs including HAD1, which shares homology with the mammalian 3-hydroxyacyl-CoA dehydrogenases. Acetyl-CoA can be further oxidized within the tricarboxylic acid cycle (TCA) to produce reduced cofactors that fuel the RC for ATP production. **b**, The inhibition of FA mitochondrial import by CPT1 (CPT1 KD) in adult MB neurons impaired memory after massed training (*n* = 18, *F*_2,51_ = 4.40 and *P* = 0.017) but not after spaced training (*n* = 13, 12, 12; *F*_2,34_ = 1.86; *P* = 0.171). **c**,**d**, The inhibition of FA mitochondrial β-oxidation in adult MB neurons by either MTPα (**c**) or HAD1 (**d**) KD impaired memory after massed training (**c**: *n* = 20, 22, 22; *F*_2,61_ = 4.43; *P* = 0.016 and **d**: *n* = 18, *F*_2,51_ = 9.43, *P* = 0.0003) but not after spaced training (**c**: *n* = 15, 15, 13; *F*_2,40_ = 1.56; *P* = 0.221 and **d**: *n* = 12, *F*_2,33_ = 0.21, *P* = 0.812). **e**, The ATP FRET sensor AT1.03NL was expressed in adult MB neurons, visualized in the CFP and YFP channels and quantified in the soma region of MB neurons (scale bar of 15 μm). Application of 5 mM of sodium azide (dashed line) resulted in a fast decrease in the FRET ratio (Δ*R*/*R*_0_), making it possible to estimate the level of ATP consumption before sodium azide application by quantifying the slope of the FRET ratio decrease. After massed associative training (green), the rate of the resulting ATP decrease was faster than in flies subjected to the unpaired protocol (black; *n* = 12, *t*_22_ = 3.06 and *P* = 0.0057). When MTPα is knocked down in adult MB neurons, the effect of associative massed training on ATP consumption was lost (*n* = 10, 11; *t*_19_ = 0.06 and *P* = 0.956). **f**–**h**, The constitutive expression of the LD-GFP transgene in MB neurons (**f**) revealed the presence of LDs in the somas of MB neurons (indicated by arrowheads; white box inset: magnified area, maximum *z*-stack projection of three confocal slices; total *z* axis of 1 µm; scale bars represent 15 µm and inset scale bars represent 4 µm). Genetically impairing FA mitochondrial import (CPT1 KD) or β-oxidation (MTPα KD) in MB neurons increased LD density in somas of MB neurons (**g**: *n* = 7, 8, 7; *F*_2,19_ = 26.51 and *P* = 0.000003), whereas the volume of individual LDs did not change (**h**: *n* = 4, 8, 7; *F*_2,16_ = 0.71 and *P* = 0.051). For ATP imaging (**e**), a representative trace of recording is shown for each condition (mean traces are shown in Extended Data Fig. 2b,d). Data on barplots are expressed as the mean ± s.e.m., with dots as individual values, and analysed by one-way ANOVA with post hoc testing by Newman–Keuls pairwise comparisons test (**b**–**d**) or by two-sided unpaired *t*-test (**e**, **g** and **h**). Genotype sample sizes are listed in the legend in the order of bar appearance. The significance level of a two-sided unpaired *t*-test (**e**, **g** and **h**) or the Newman–Keuls pairwise comparison between the genotype of interest and the genotypic controls (**b**–**d**) following one-way ANOVA is as follows: *P < 0.05, ***P < 0.001 and ****P < 0.0001. NS, not significant. Source data

**Extended Data Fig. 1:**
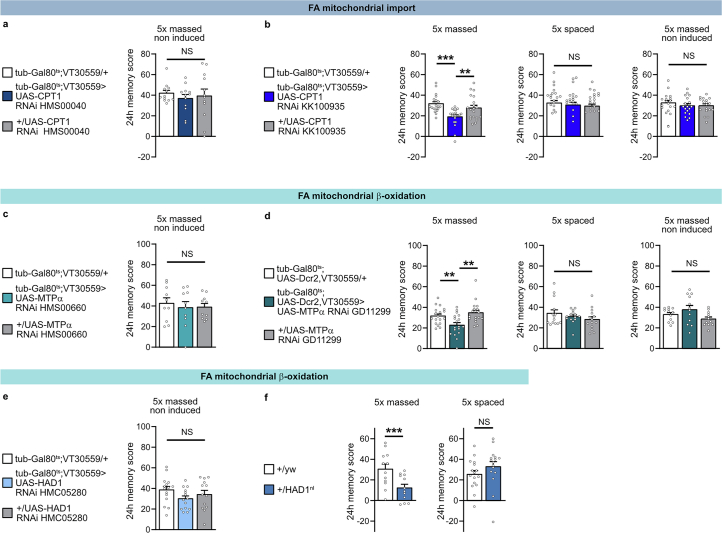
, related to Fig. 1: Control experiments for FA mitochondrial import and β-oxidation in MB neurons to sustain memory formed after massed training. Extended Data Fig. 1**a**. When CPT1 RNAi expression was not induced, memory after massed training was normal (n = 12, F_2,33_ = 0.30, P = 0.743). **b**. A second non-overlapping RNAi targeting CPT1 (CPT1 RNAi KK100935) was used to confirm the specific defect in memory after massed training. Inhibition of CPT1 expression in adult MB neurons using CPT1 RNAi KK100935 impaired memory after massed training (n = 20, F_2,57_ = 8.91, P = 0.0004), whereas memory after spaced training was normal (n = 18, F_2,51_ = 0.43, P = 0.656). Non-induced flies showed no memory defect after massed training (n = 18, F_2,51_ = 0.61, P = 0.546). **c**. When MTPα RNAi expression was not induced, memory after massed training was normal (n = 10, F_2,27_ = 0.21, P = 0.816). **d**. A second non-overlapping RNAi targeting MTPα (MTPα RNAi GD11299) was used to confirm the specific defect in memory after massed training. In order to increase RNAi efficiency, Dicer2 expression was induced together with the RNAi expression in adult MB neurons using the Tub-Gal80ts; UAS-Dcr2, VT30559 line. Inhibition of MTPα expression in adult MB neurons using this RNAi impaired memory after massed training (n = 18, F_2,51_ = 7.30, P = 0.0016), whereas memory after spaced training was normal (n = 14, F_2,39_ = 1.21, P = 0.309). Non-induced flies showed no memory defect after massed training (n = 12, F_2,33_ = 2.62, P = 0.088). **e**. When HAD1 RNAi expression was not induced, memory after massed training was normal (n = 13-15, F_2,39_ = 1.63, P = 0.209). **f**. Because only one RNAi line targeting Had1 was available, we used a HAD1 null mutant to confirm HAD1 function in memory formed after massed training, albeit without cell-type specificity. Since HAD1^nl^ is in a yw background, we used heterozygous +/yw flies as a control for the +/HAD1^nl^ experimental group. Heterozygous +/HAD1^nl^ flies had a memory defect after massed training as compared to control +/yw flies (n = 12, t_22_ = 2.97, P = 0.0071), whereas memory after spaced training was normal (n = 16, t_30_ = 1.23, P = 0.229). Data on barplots are expressed as mean ± s.e.m. with dots as individual values, and analyzed by ANOVA with post hoc testing by Newman–Keuls pairwise comparisons test (a-e) or by two-sided unpaired t-test (f). Genotype sample sizes are listed in the legend in order of bar appearance. Significance level of a two-sided unpaired t-test (f) or the Newman–Keuls pairwise comparison between the genotype of interest and the genotypic controls (a-e) following one-way ANOVA: **P < 0.01, ***P < 0.001, NS: not significant. Source data

To test this hypothesis, we first targeted the system that imports FAs into the mitochondria. Long-chain FAs must first be activated to acyl-coenzyme A (acyl-CoA) esters by cytosolic acyl-CoA synthetase before they can be directed into the mitochondria by the carnitine shuttle system^18,19^ (Fig. 1a). Since the outer mitochondrial membrane component of this shuttle, carnitine palmitoyltransferase 1 (CPT1), catalyses the rate-limiting step of FA import^20^, we tested its involvement in memory using an RNA interference (RNAi)-based knock-down (KD). To restrict the expression of CPT1 RNAi to adult MB neurons, we used the MB-specific VT30559-Gal4 driver^14^ in combination with the ubiquitously expressed thermosensitive Gal4 inhibitor, Gal80 (tub-Gal80^ts^)^21^. Placing adult flies at 30 °C for 2–3 days therefore allows RNAi expression in MB neurons (see Methods for details). Flies were then subjected to massed training and tested for memory performance 24 h later. CPT1 KD in adult MB neurons impaired memory after massed training, whereas 24-h memory formed after the spaced training was normal (Fig. 1b). Memory after massed training was normal in a control experiment in which CPT1 RNAi expression was not induced (Extended Data Fig. 1a). Finally, CPT1 KD did not alter innate shock reactivity or olfactory acuity (Supplementary Table 1). These results were replicated with a second non-overlapping RNAi line targeting CPT1 (Extended Data Fig. 1b and Supplementary Table 1). For simplicity, the combined phenotypes described above are hereafter described as a specific defect in memory after massed training. Both CPT1 RNAi lines, previously validated in glial cells^17^, efficiently reduced the CPT1 messenger RNA level in *Drosophila* brains when expressed specifically in neurons (Supplementary Table 2). Once inside the mitochondria, activated FAs can be oxidized into acetyl-CoA via the β-oxidation pathway, a cyclic process that generates reduced NADH and FADH_2_ and requires the successive action of different chain length-specific enzymes for the complete oxidation of FAs^19,22^ (Fig. 1a). We therefore investigated the role of two major enzymes in this pathway: the mitochondrial trifunctional protein (MTP), which shortens long-chain FAs to medium-chain lengths^22,23^, and HAD1, an enzyme more specific to short-chain FAs, with homology to mammalian 3-hydroxyacyl-CoA dehydrogenases^22,24^. Downregulation of either MTPα (the α subunit of MTP) or HAD1 expression in adult MB neurons caused a specific memory defect after massed training (Fig. 1c,d, Extended Data Fig. 1c–f and Supplementary Tables 1 and 2). Downregulation of either CPT1, MTPα or HAD1 in MB neurons resulted in reduction of mRNA levels of the target gene in *Drosophila* brain (Supplementary Table 3), validating that these genes are expressed in MB neurons. Altogether, these data show that FA mitochondrial import and their subsequent β-oxidation are required in MB neurons to sustain memory formation after massed training.

**Extended Data Fig. 2:**
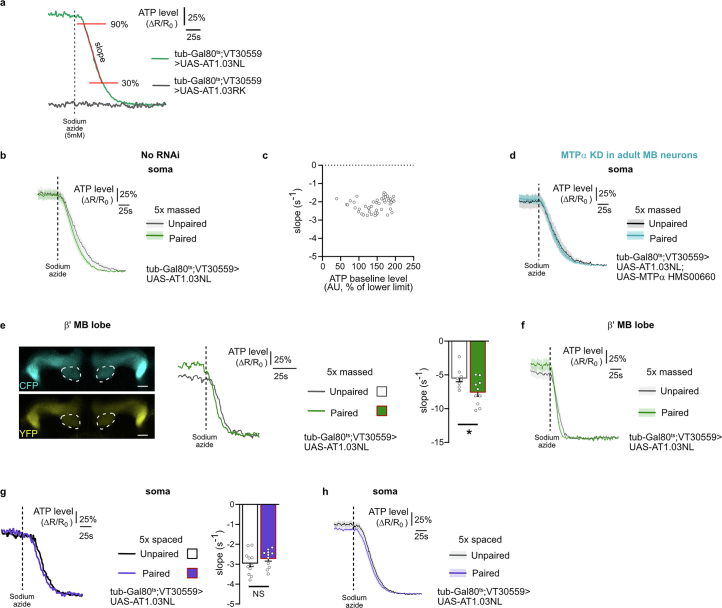
, related to Fig. 1 Control experiments for ATP imaging experiments. Extended Data Fig. 2**a**. Applying 5 mM of sodium azide (dashed line) resulted in a fast decrease in the FRET ratio of the ATP FRET sensor AT1.03NL expressed in adult MB neurons, whereas the FRET ratio of AT1.03RK (a nonfunctional version of the probe that is ATP insensitive) did not change (mean trace ± s.e.m.). The slope of the FRET ratio was measured between 90% and 30% of the baseline level (plateau reached after sodium azide application corresponding to the lower limit of the FRET sensor detection). **b**. related to Fig. 1e: Mean traces of ATP consumption of MB neurons of flies subjected to either associative massed training (green) or the unpaired protocol (black). **c**. XY-plot of individual fly datapoints of baseline ATP level and the corresponding slopes of all control flies (No RNAi and subjected to unpaired massed protocol) showing no correlation between ATP baseline level and the rate of ATP decrease (the slope) triggered by sodium azide application (Pearson correlation: n = 49, R² = 0.06, p = 0.09). **d**. related to Fig. 1e: Mean traces of ATP consumption of MB neurons KD for MTPα of flies subjected to either massed associative training (blue) or the unpaired protocol (black). **e**. In the β’2 subdomain of the β’ lobes (Region of recording of MB neurons outline in white dotted line, scale bar: 15 μm), massed associative training (green) triggered an increase of ATP consumption compared to flies subjected to the unpaired protocol (black; n = 8,10, t_16_ = 2.49, P = 0.0243), a representative trace of recording is presented for each condition **f**. Mean traces of ATP consumption recorded in the β’2 subdomain of MB neurons of flies subjected to either massed associative training (green) or the unpaired (black). **g**. In the soma of MB neurons, no difference in ATP consumption was observed after spaced training (purple: associative, black: non-associative protocol, n = 11, t_20_ = 1.06, P = 0.304), a representative trace of recording is presented for each condition. **h**. Mean traces of ATP consumption recorded in the soma of MB neurons of flies subjected to either spaced associative training (purple) or the non-associative spaced protocol (black). Data on barplots are expressed as mean ± s.e.m. with dots as individual values, and analyzed by two-sided unpaired t-test (e,g). Genotype sample sizes are listed in the legend in order of bar appearance. Significance level of a two-sided unpaired t-test (e-g): *P < 0.05, **P < 0.01, ***P < 0.001, NS: not significant. Source data

Since β-oxidation is associated with ATP production^22^, we investigated whether ATP production in MB neurons was increased following massed training, as compared with a control, non-associative massed protocol, in which the presentations of electric shocks and odour are dissociated in time (unpaired protocol). To image ATP levels in vivo, we expressed in MB neurons the genetically-encoded ATP fluorescence resonance energy transfer (FRET) sensor AT1.03NL, a modified version of the widely used ATeam ATP sensor, which is optimized for use in non-homeothermic species such as *Drosophila*^25^. Following sodium azide application, which blocks the mitochondrial respiratory chain, the FRET ratio of the ATP sensor showed a rapid drop, the slope of which can be used to estimate the level of ATP consumption (Extended Data Fig. 2a). After massed training, the rate of sodium azide-induced ATP decrease was higher than that observed in flies subjected to the unpaired protocol, indicating a higher consumption of ATP produced by mitochondria in the soma of MB neurons (Fig. 1e and Extended Data Fig. 2b–d). This effect was also observed in the β′2 synaptic compartment, the synaptic site involved in memory retrieval after massed training^26^ (Extended Data Fig. 2e,f). When MTPα was knocked down in adult MB neurons, the effect of massed training on ATP consumption was lost (Fig. 1e), showing that β-oxidation sustains the increased ATP production in soma of MB neurons triggered by massed training. Interestingly, this increased ATP consumption was observed after massed training but not after spaced training (Extended Data Fig. 2g,h), in agreement with behavioural analyses.

**Extended Data Fig. 3:**
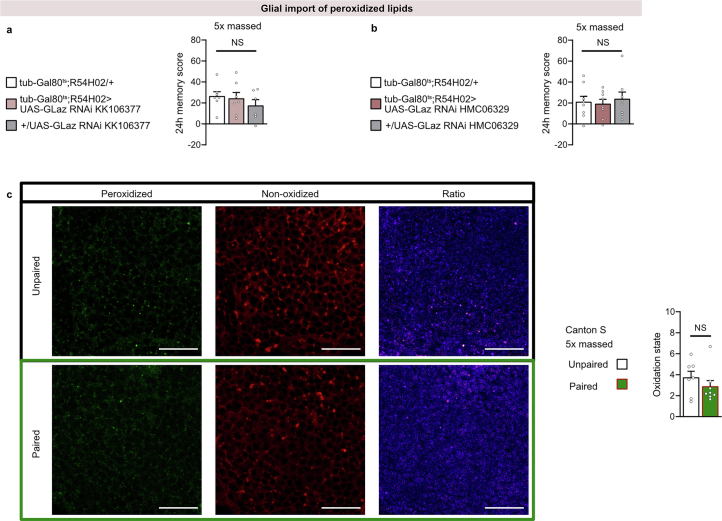
, related to Fig. 1: Lipid oxidation level. Extended Data Fig. 3**a-b**. Inhibition of glial import of oxidized lipid via GLaz in adult cortex glia using two different RNAi had no effect on memory after massed training (**a**, n = 7,7,6, F_2,17_ = 0.69, P = 0.515, **b**. n = 8, F_2,21_ = 0.17, P = 0.842,) **c**. Lipid oxidation state in the soma region of MB neurons measured by C11-BODIPY^581/591^ (labelling both cell membrane and lipid droplets of cortex glia and neurons) was not affected by associative massed training as compared to unpaired controls (n = 7,8, t_13_ = 0.99, P = 0.339). scale bar: 15 µm. Data on barplots are expressed as mean ± s.e.m. with dots as individual values, and analyzed by ANOVA with post hoc testing by Newman–Keuls pairwise comparisons test (**a**-**b**) or by two-sided unpaired t-test (**c**). Genotype sample sizes are listed in the legend in order of bar appearance. Significance level of a two-sided unpaired t-test (**c**) or the Newman–Keuls pairwise comparison between the genotype of interest and the genotypic controls (**a**-**b**) following one-way ANOVA: NS: not significant. Source data

FA β-oxidation is often associated with the production of mitochondrial reactive oxygen species (ROS) resulting in toxic peroxidized lipid accumulation. We investigated whether the FA β-oxidation occurring in MB neurons during memory formation after massed training increased peroxidized lipid production. To avoid accumulation of peroxidized lipids in neurons, cortex glia, which surround MB neuron somas in a honeycomb-like network of glial process^27^, can import peroxidized lipids via the GLaz transporter and store them in lipid droplets (LDs)^28,29^. However, GLaz KD in adult cortex glia using the specific cortex glia driver R54H02, combined with tub-Gal80^ts^ (ref. ^30^), had no impact on memory formed after massed training (Extended Data Fig. 3a,b). In addition, using C11-BODIPY^581/591^ to measure the peroxidation level of lipids^29^, we did not observe any increase in peroxidized lipid levels after massed training in the region encompassing both MB neuron somas and cortex glia processes (Extended Data Fig. 3c). Altogether these results suggest that after massed training, MB neurons are able to cope with putative ROS production linked to FA oxidation.

To further support our finding that FA β-oxidation occurs in vivo in MB neurons, we investigated whether the impaired import of FAs to mitochondria and their subsequent oxidation results in FA accumulation. Because free FAs in the cytoplasm are toxic^31^, they are stored in LDs^32^. To reveal the accumulation of FA, we used an LD-green fluorescent protein (LD-GFP) transgene^9,33^. In flies, when constitutively expressing the LD marker LD-GFP under the control of a specific MB neuronal driver, only a few LDs were detected in the soma of MB neurons, as previously described^9^. In contrast, genetically impairing FA mitochondrial import (CPT1 KD) or β-oxidation (MTPα KD) in MB neurons led to the abnormal accumulation of LDs in their somas (Fig. 1f–h). These results show that impairing FA import and oxidation by mitochondria in MB neurons results in their intracellular accumulation.

**Fig. 2:**
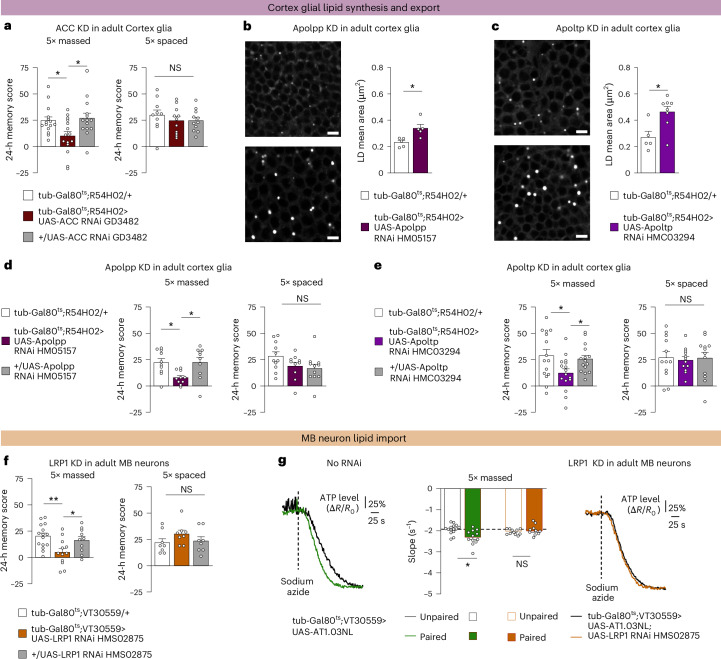
Cortex glia provide lipids to MB neurons to sustain memory formation after massed training. **a**, Genetically impairing lipogenesis (ACC KD) in adult cortex glia impaired memory formation after massed training (*n* = 15, 15, 14; *F*_2,41_ = 4.7; *P* = 0.014) but not after spaced training (*n* = 10, 11, 11; *F*_2,29_ = 0.54; *P* = 0.591). **b**,**c**, The inhibition of Apolpp (**b**) or Apoltp (**c**) expression in adult cortex glia resulted in larger LDs measured in the region of MB soma by BODIPY staining compared with controls (Apolpp: *n* = 5, *t*_8_ = 3.22, *P* = 0.012 and Apoltp: *n* = 5, 8; *t*_11_ = 2.98; *P* = 0.013). Scale bars, 4 µm. **d**,**e**, Genetically impairing apolipoprotein formation (**d**, Apolpp KD) or maturation (**e**, Apoltp KD) in adult cortex glia impaired memory formation after massed training (Apolpp: *n* = 10, *F*_2,27_ = 5.1, *P* = 0.013; Apoltp: *n* = 15, 16, 15; *F*_2,43_ = 3.9; *P* = 0.029) but not after spaced training (Apolpp: *n* = 11, 10, 11; *F*_2,29_ = 2.4, *P* = 0.107; Apoltp: *n* = 12, *F*_2,33_ = 0.06, *P* = 0.945). **f**, Genetically impairing apolipoprotein import (LRP1 KD) in adult MB neurons impaired memory formation after massed training (*n* = 15, 12, 10; *F*_2,34_ = 5.8; *P* = 0.007) but not after spaced training (*n* = 8, *F*_2,21_ = 1.31, *P* = 0.291). **g**, Massed training (green) triggered an increase of ATP consumption as compared with flies subjected to the unpaired protocol (black; *n* = 14, 12; *t*_24_ = 3.34; *P* = 0.0027). LRP1 KD in adult MB neurons abolished the effect of massed training on ATP consumption (*n* = 11, 10; *t*_19_ = 0.99; *P* = 0.336). For ATP imaging (**g**), a representative trace of recording is presented for each condition (the mean traces are shown in Extended Data Fig. 4e,f). Data on barplots are expressed as the mean ± s.e.m., with dots as individual values, and analysed by one-way ANOVA with post hoc testing by Newman–Keuls pairwise comparisons tests (**a** and **d**–**f**) or by two-sided unpaired *t*-tests (**b**, **c** and **g**). Genotype sample sizes are listed in the legend in the order of bar appearance. The significance levels of two-sided unpaired *t*-tests (**b**, **c** and **g**) or the Newman–Keuls pairwise comparisons between the genotype of interest and the genotypic controls (**a** and **d**–**f**) following one-way ANOVA are *P < 0.05 and **P < 0.01. NS, not significant. Source data

**Extended Data Fig. 4:**
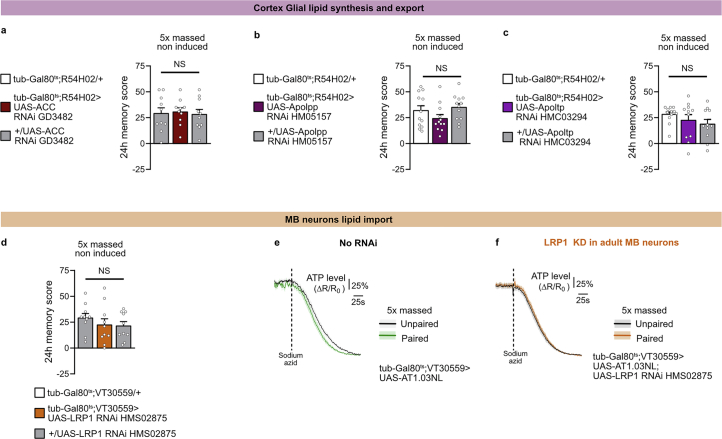
, related to Fig. 2: Control experiments for lipids transfer from cortex glia to MB neurons required to sustain memory formed after massed training. Extended Data Fig. 4**a**. When ACC RNAi expression was not induced, memory after massed training was normal (n = 10, F_2,27_ = 0.06, P = 0.939). **b**. When Apolpp RNAi expression was not induced, memory after massed training was normal (n = 12, F_2,33_ = 1.90, P = 0.170). **c**. When Apoltp RNAi expression was not induced, memory after massed training was normal (n = 12,11,11, F_2,31_ = 1.30, P = 0.289). **d**. When LRP1 RNAi expression was not induced, memory after massed training was normal (n = 10, F_2,27_ = 0.72, P = 0.494). **e**. related to Fig. 2g: Mean traces of ATP consumption of MB neurons of flies subjected to either massed associative training (green) or the unpaired protocol (black). **f**. related to Fig. 2g: Mean traces of ATP consumption of MB neurons KD for LRP1 of flies subjected to either massed associative training (orange) or the unpaired protocol (black). Data on barplots are expressed as mean ± s.e.m. with dots as individual values, and analyzed by ANOVA with post hoc testing by Newman–Keuls pairwise comparisons test (**a**-**d**). Genotype sample sizes are listed in the legend in order of bar appearance. Significance level of the Newman–Keuls pairwise comparison between the genotype of interest and the genotypic controls (**a**-**d**) following one-way ANOVA: NS: not significant. Source data

We then investigated whether the FAs could be provided by the glia surrounding the MB neurons cell bodies, that is, the cortex glia. To address this question, we first investigated lipogenesis by targeting the limiting step of FA synthesis, which involves the enzyme acetyl-CoA carboxylase (ACC)^34^. ACC KD in adult cortex glia induced a specific memory impairment after massed training (Fig. 2a, Extended Data Fig. 4a and Supplementary Tables 2 and 4). Intercellular transfer of lipids requires their association to transport proteins, forming lipoprotein systems^35^. Production of lipoprotein particles relies on scaffold proteins onto which lipids are loaded^35^. Apolpp and Apoltp, the two majors lipoprotein scaffolds in *Drosophila*, act in a sequential process to form mature lipoprotein particles and mediate interactions with lipoproteins receptors to facilitate lipid uptake by neurons^35–37^. When Apolpp or Apoltp expression is knocked down in adult cortex glia, we observed an increase of LD size as a probable consequence of lipid intracellular accumulation (Fig. 2b,c). These results suggest that both Apolpp and Apoltp are required in cortex glia for lipid export. We then investigated whether lipid export from cortex glia is required for memory formation after massed training. We observed that Apolpp KD as well as Apoltp KD in adult cortex glia caused specific defects in memory formation after massed training (Fig. 2d,e, Extended Data Fig. 4b,c and Supplementary Tables 2 and 4). Altogether, these results show that lipogenesis and lipoprotein particle production by cortex glia are two processes required for memory formation after massed training.

Lipoprotein particle uptake by cells involves specific proteins. As the low-density lipoprotein receptor-related protein 1 (LRP1) receptor is strongly expressed in MB neurons^38,39^, we investigated its role in lipid import for memory formation. LRP1 KD in adult MB neurons induced a specific memory impairment after massed training and abolished the ATP production increase triggered by massed training (Fig. 2f,g, Extended Data Fig. 4d–f and Supplementary Tables 2 and 4). Altogether, our data depict a transfer of lipids from cortex glia to MB neurons via lipoprotein particle uptake by LRP1, fuelling ATP production to sustain memory formation after massed training.

**Fig. 3:**
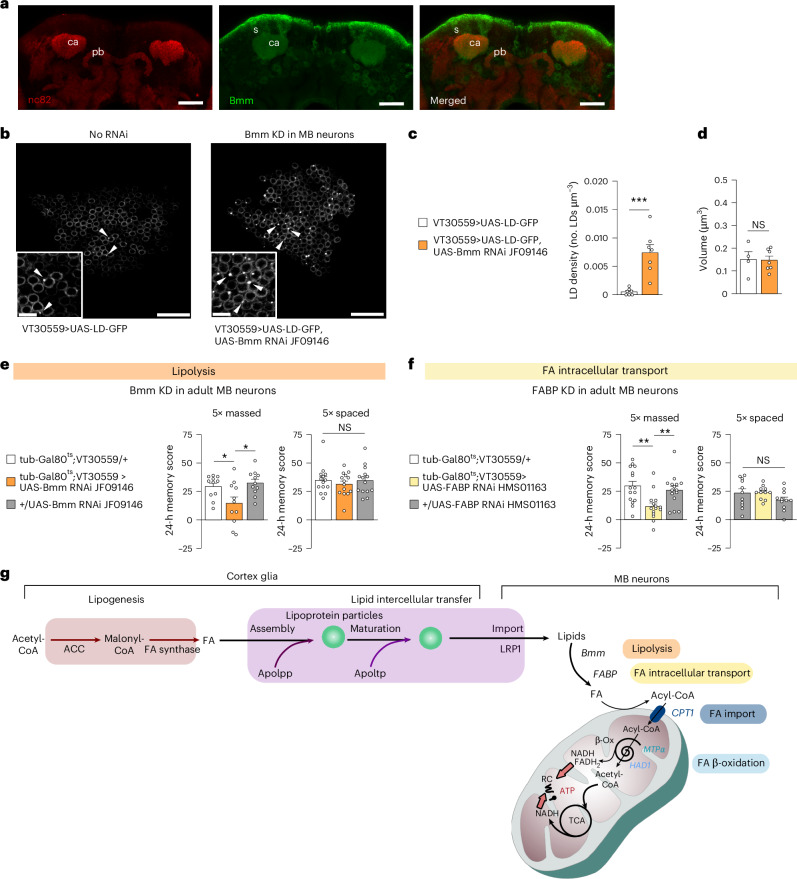
In MB neurons, Bmm lipolysis and FABP-mediated intracellular FA transport are required for memory formation after massed training. **a**, Immunohistochemistry of Mi{Trojan-GAL4.1}bmm^MI13321-TG4.1^>UAS-mCD8::GFP brains showing the Bmm expression pattern in green (GFP) and the pan-neuronal anti-nc82 counterstaining in red. Images are a maximum intensity projection of three confocal planes (total *z* axis of 2 µm) of the soma and calyx MB region of the *Drosophila* brain (posterior brain). Clear Bmm expression can be detected in the soma and the calyx of MB neurons. Scale bar, 30 µm. **b**–**d**, Genetically impairing lipolysis (Bmm KD) in MB neurons (**b**) increased LD density in the somas of MB neurons compared with the genotypic control (**c**, *n* = 7, *t*_12_ = 4.78 and *P* = 0.0004), whereas the volume of individual LDs did not change (**d**, *n* = 4,7, *t*_9_ = 0.09, *P* = 0.93). LDs are indicated by arrowheads and the white box insets represent magnified areas of maximum *z*-stack projections of three confocal slices (total *z* axis of 1 µm). Scale bars represent 15 µm, and inset scale bars represent 4 µm. **e**, Bmm KD in adult MB neurons impaired memory formation after massed training (*n* = 11, *F*_2,30_ = 5.33 and *P* = 0.011) but not after spaced training (*n* = 14, *F*_2,39_ = 0.36 and *P* = 0.702). **f**, Genetically impairing FA intracellular transport (FABP KD) in adult MB neurons impaired memory formation after massed training (*n* = 15, *F*_2,42_ = 7.22 and *P* = 0.002) but not after spaced training (*n* = 11, *F*_2,30_ = 1.74 and *P* = 0.192). **g**, A schema of the identified actors of FA metabolism required for memory formation after massed training. Please note that apolipoprotein maturation by Apoltp can take place either inside cortex glia or in the extracellular space. Data on barplots are expressed as the mean ± s.e.m., with dots as individual values, and analysed by one-way ANOVA with post hoc testing by Newman–Keuls pairwise comparisons tests (**e** and **f**) or by two-sided unpaired *t*-test (**c** and **d**). Genotype sample sizes are listed in the legend in order of bar appearance. The significance levels of a two-sided unpaired *t*-test (**c** and **d**) or the Newman–Keuls pairwise comparisons between the genotype of interest and the genotypic controls (**e** and **f**) following one-way ANOVA are *P < 0.05, **P < 0.01 and ***P < 0.001. s, soma of MB neurons; ca, calyx of MB; pb, protocerebral bridge of the central complex; NS, not significant. Source data

**Extended Data Fig. 5:**
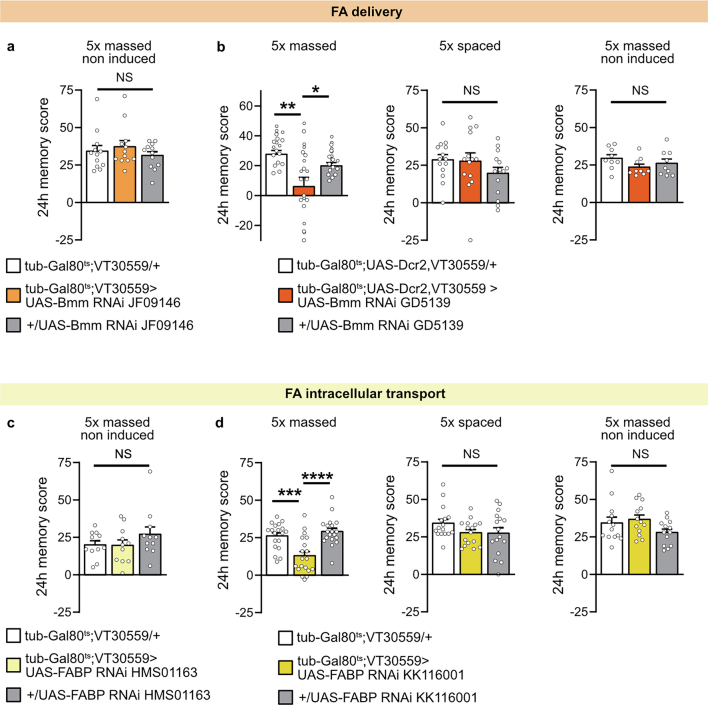
, related to Fig. 3: Control experiments for intracellular FA delivery in MB neurons to sustain memory formed after massed training. Extended Data Figure 5**a**. When Bmm RNAi expression was not induced, memory after massed training was normal (n = 12, F_2,33_ = 0.67, P = 0.518). **b**. A second non-overlapping RNAi targeting Bmm (Bmm RNAi GD5139) was used to confirm the specific defect in memory after massed training. As previously carried out in^17^ using this RNAi line, Dicer2 expression was induced together with RNAi expression in adult MB neurons (Tub-Gal80ts; UAS-Dcr2, VT30559 line) to increase RNAi efficiency. Bmm KD in adult MB neurons impaired memory after massed training (n = 12, F_2,33_ = 7.21, P = 0.0025), whereas memory after spaced training was normal (n = 14, F_2,39_ = 1.24, P = 0.300). Non-induced flies showed no memory defect after massed training (n = 9, F_2,24_ = 1.50, P = 0.244). **c**. When FABP RNAi expression was not induced, memory after massed training was normal (n = 11, F_2,30_ = 1.15, P = 0.329). **d**. A second non-overlapping RNAi targeting FABP (FABP RNAi KK116001) was used to confirm the specific defect in memory after massed training. FABP KD in adult MB neurons impaired memory after massed training (n = 19, F_2,54_ = 13.51, P = 0.00002), whereas memory after spaced training was normal (n = 16, F_2,45_ = 1.72, P = 0.190). Non-induced flies showed no memory defect after massed training (n = 13, F_2,36_ = 2.12, P = 0.135). Data on barplots are expressed as mean ± s.e.m. with dots as individual values, and analyzed by ANOVA with post hoc testing by Newman–Keuls pairwise comparisons test (**a**-**d**). Genotype sample sizes are listed in the legend in order of bar appearance. Significance level of the Newman–Keuls pairwise comparison between the genotype of interest and the genotypic controls (**a**-**d**) following one-way ANOVA: *P < 0.05, **P < 0.01, ***P < 0.001, ****P < 0.0001 NS: not significant. Source data

Once inside the MB neurons, lipids need to be hydrolysed into FAs and delivered to the mitochondria. Among several lipases, Brummer (Bmm), the orthologue of mammalian adipose triglyceride lipase (ATGL)^40^, is of particular interest as it is expressed in MB neurons (Fig. 3a). Bmm KD in MB neurons resulted in LD accumulation (Fig. 3b–d). Remarkably, Bmm KD in adult MB neurons resulted in a specific memory impairment after massed training (Fig. 3e, Extended Data Fig. 5a,b and Supplementary Tables 3 and 5). To limit free FA toxicity^31^, FAs are transported to mitochondria in association with FA-binding proteins (FABPs)^41,42^. We observed that FABP KD in adult MB neurons caused a specific memory defect after massed training (Fig. 3f, Extended Data Fig. 5c,d and Supplementary Tables 2, 3 and 5). On the basis of these data, we propose that, to support memory formation following cramming-like learning, a glia-to-neuron FA flux is at play, from the production of FAs by cortex glia to their usage by neurons for mitochondrial energy production (Fig. 3g).

**Fig. 4:**
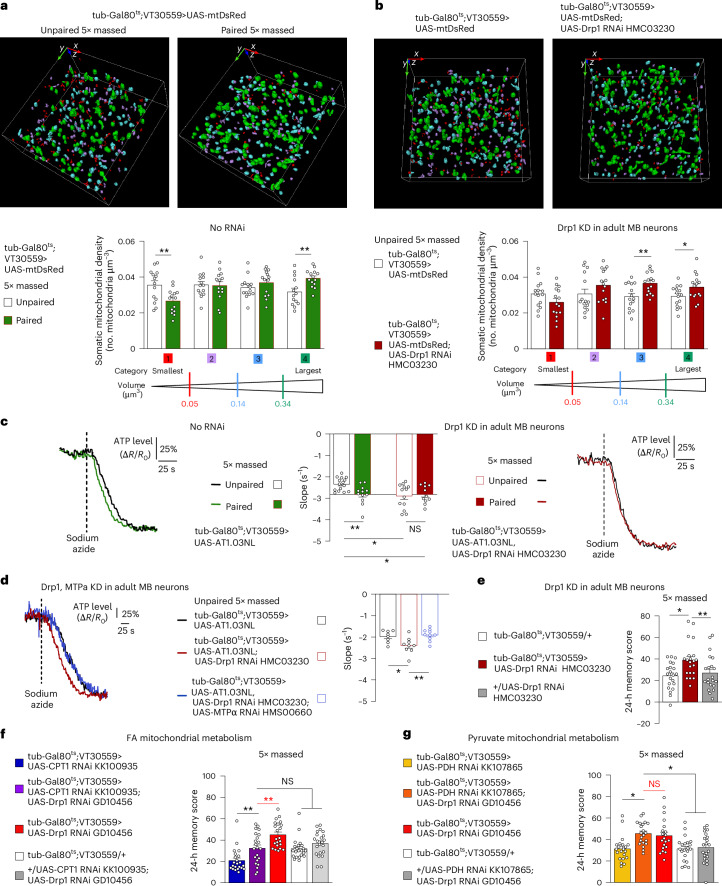
Mitochondrial network remodelling facilitates ATP production through FA oxidation in MB neurons and improves memory after massed training. **a**, Mitochondria were categorized according to their volume into four groups. Detected mitochondria are shown with their colour-coded categories (the smallest mitochondria as category 1, red; small intermediate as category 2, purple; medium intermediate as category 3, blue; and the largest mitochondria as category 4, green). Three-dimensional co-ordinate axes indicate 2.5 µm in each direction. To compare between the different conditions, we calculated the density of mitochondria for each category by normalizing the number of mitochondria in each category by the volume of the minimal envelope containing all of the detected mitochondria in the ROI (see Methods for details). Subsequently, 1 h after training, in flies subjected to 5× massed associative training, the density of the largest mitochondria was increased in MB somas compared with control flies subjected to the unpaired protocol (largest mitochondria, category 4: *n* = 14, *t*_26_ = 3.08 and *P* = 0.0049). The intermediate categories (2 and 3) were not significantly affected by associative massed training (intermediate mitochondria, category 2: *n* = 14, *t*_26_ = 0.15 and *P* = 0.883 and category 3: *n* = 14, *t*_26_ = 1.12 and *P* = 0.275), whereas the density of the smallest mitochondria was decreased upon associative training (smallest mitochondria, category 1: *n* = 14, *t*_26_ = 3.01 and *P* = 0.0057). **b**, When Drp1 was knocked down in adult MB, the density of large mitochondria in MB neuronal somas was increased as compared with the genotypic control (category 3: *n* = 15, *t*_28_ = 3.11 and *P* = 0.0043 and category 4: *n* = 15, *t*_28_ = 2.17 and *P* = 0.0391), whereas the density of small mitochondria did not change (category 1: *n* = 15, *t*_28_ = 1.64 and *P* = 0.113 and category 2: *n* = 15, *t*_28_ = 1.34 and *P* = 0.193). **c**, Massed training (green) triggered an increase of ATP consumption as compared with flies subjected to the unpaired protocol (black). When Drp1 was knocked down in adult MB neurons, ATP consumption was increased as compared with wild-type flies subjected to unpaired training (*n* = 15, 12, 13, 11; *F*_3,47_ = 4.60 and *P* = 0.0067. The *P* values of relevant pairwise comparisons are indicated). **d**, The increased ATP consumption in flies with Drp1 KD in MB adult neurons was abolished by concurrent MTPα KD (*n* = 8, 9, 10; *F*_2,24_ = 6.48; *P* = 0.0056. The *P* values of relevant pairwise comparisons are indicated). **e**, Drp1 KD in adult MB neurons increased memory performance after massed training (*n* = 21, *F*_2,60_ = 5.55 and *P* = 0.0061). **f**,**g**, The increase of memory performance observed after massed training in Drp1 KD flies was abolished by CPT1 KD (**f**, *n* = 22, *F*_4,105_ = 12.29, *P* = 0.00000003; the *P* values of relevant pairwise comparisons are indicated; the red statistical marker shows the comparison between Drp1 KD and CPT1, Drp1 KD flies) and preserved when mitochondrial pyruvate metabolism was impaired (PDH KD) (**g**, *n* = 21, 21, 20, 21, 21; *F*_4,99_ = 6.71; *P* = 0.00008; the *P* values of relevant pairwise comparisons are indicated; the red statistical marker shows the comparison between Drp1 KD and PDH, Drp1 KD flies). For ATP imaging (**c** and **d**), a representative trace of recording is presented for each condition (mean traces are in Extended Data Fig. 6b–d). Data on barplots are expressed as the mean ± s.e.m., with dots as individual values, and analysed for mitochondria volume by two-sided unpaired *t*-tests (**a** and **b**) and for ATP consumption experiments and behaviour experiments by ANOVA with post hoc testing by Newman–Keuls pairwise comparisons tests (**c**–**g**). Genotype sample sizes are listed in the legend in the order of bar appearance. The significance levels of two-sided unpaired *t*-tests (**a** and **b**) or the Newman–Keuls pairwise comparisons between the genotype of interest and the genotypic controls (**c**–**g**) following one-way ANOVA are *P < 0.05 and **P < 0.01. NS, not significant. Source data

**Extended Data Fig. 6:**
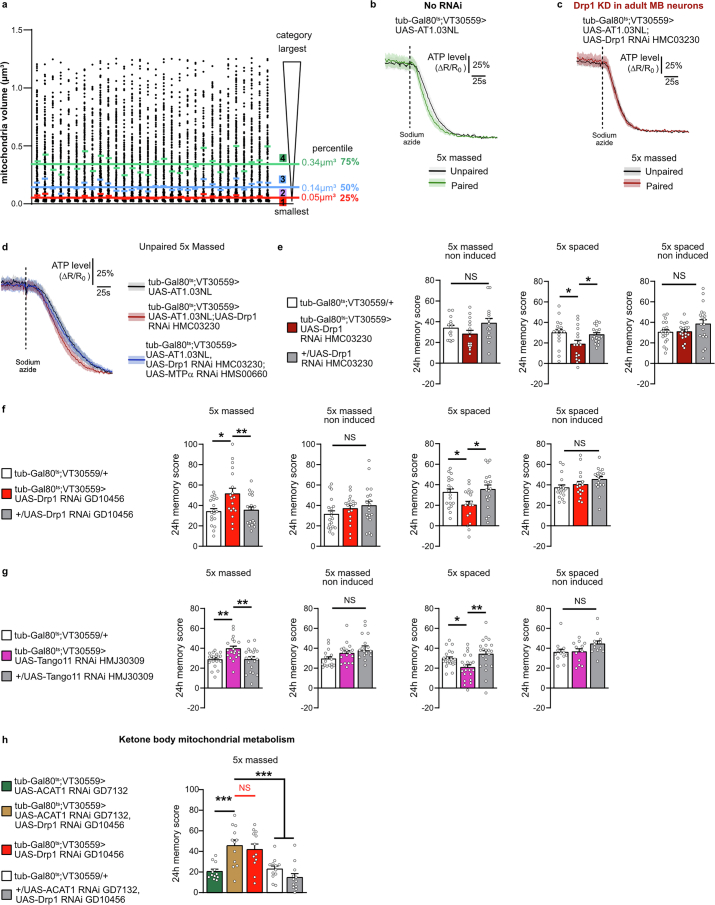
, related to Fig. 4: Control experiments for mitochondrial network remodeling facilitates ATP production in the soma of MB neurons and improves memory after massed training. Extended Data Figure 6**a**. The mitochondria volume distribution is shown for each ROI of all unpaired control flies used in this study, where each dot represents the volume of one single identified mitochondrion. The means of the quartile limits from all control flies subjected to the massed unpaired protocol (*that is* tub-Gal80^ts^; VT30559 > UAS-mtDsRed flies subjected to the unpaired 5x massed protocol in Fig. 4a-b) were used to define the 4 mitochondria categories. **b-c**. related to Fig. 4c. **b:** Mean traces of ATP consumption of MB neurons of flies subjected to either massed associative training (green) or the unpaired protocol (black). **c**: Mean traces of ATP consumption of MB neurons KD for Drp1 of flies subjected to either massed associative training (red) or the unpaired protocol (black). **d** related to Fig. 4d: Mean traces of ATP consumption of MB neurons of flies subjected to unpaired protocol (red: Drp1 KD flies, blue: Drp1, MTPa KD flies, black: No RNAi flies)**. e**. When Drp1 RNAi expression was not induced, memory after massed training was normal (n = 15, F_2,42_ = 2.01, P = 0.147). Drp1 KD in adult MB neurons impaired memory after spaced training (n = 17, F_2,48_ = 3.68, P = 0.0325). When Drp1 RNAi expression was not induced, memory after spaced training was normal (n = 19, F_2,54_ = 2.25, P = 0.116). **f**. A second non-overlapping RNAi targeting Drp1 (Drp1 RNAi GD10456) was used to confirm the specific increase in memory after massed training. Inhibition of Drp1 expression in adult MB neurons using this RNAi increased memory performance after massed training (n = 18, F_2,51_ = 5.53, P = 0.0067), whereas memory after spaced training was impaired (n = 18, F_2,51_ = 4.38, P = 0.0176). Non-induced flies showed normal memory performance after either massed training (n = 19,18,19, F_2,53_ = 1.33, P = 0.274) or spaced training (n = 17, F_2,48_ = 2.01, P = 0.146). **g**. Tango11 KD in adult MB neurons increased memory performance after massed training (n = 19, F_2,54_ = 6.93, P = 0.0021), whereas memory after spaced training was impaired (n = 21, F_2,60_ = 6.05, P = 0.0040). Non-induced flies showed normal memory performance after either massed training (n = 16,17,17, F_2,47_ = 1.49, P = 0.236) or spaced training (n = 12, F_2,33_ = 2.28, P = 0.118). **h**. The increase of memory performance observed after massed training in flies KD for Drp1 in adult MB neurons was preserved when ketone body metabolism was impaired (Drp1, ACAT1 KD) (n = 12, F_4,55_ = 10.63, P = 0.000002, the p-value of the relevant pairwise comparisons are indicated). The red statistical marker shows the comparison between Drp1 KD and ACAT1, Drp1 KD flies. Data on barplots are expressed as mean ± s.e.m. with dots as individual values, and analyzed by ANOVA with post hoc testing by Newman–Keuls pairwise comparisons test (**e**-**h**). Genotype sample sizes are listed in the legend in order of bar appearance. Significance level of the Newman–Keuls pairwise comparison between the genotype of interest and the genotypic controls (**e**-**h**) following one-way ANOVA: *P < 0.05, **P < 0.01, ***P < 0.001, NS: not significant. Source data

Interestingly, it has been shown in vitro that, under specific conditions such as starvation, in which fibroblasts become more reliant on FA β-oxidation, mitochondria are remodelled into highly connected networks, and these elongated mitochondria are required for appropriate FA flux to mitochondria^43^. Thus, we asked whether mitochondria network remodelling is also involved in supporting memory formation after massed training. We first investigated whether massed training triggers mitochondrial network remodelling. We used super-resolution three-dimensional stimulated emission depletion (3D-STED) microscopy to image the mitochondria of flies expressing a DsRed fluorophore localised to the mitochondrial matrix in MB neurons, as in our previous work^44^. Briefly, we defined four categories of mitochondria on the basis of their volume and the density of mitochondria in the soma of MB neurons in each category (number of mitochondria per cubic micrometre) was compared between experiments (see Methods and Extended Data Fig. 6a for details). When flies were subjected to massed training, the density of the largest mitochondria increased in MB somas as compared with control flies subjected to the unpaired protocol (Fig. 4a), while the density of intermediate mitochondria remained unchanged and the density of the smallest mitochondria was reduced. Hence, massed training triggers mitochondrial network remodelling in the MB soma.

Mitochondria re-arrangement resembling those reported here were previously observed in MB somas after spaced training, attributable to increased mitochondria motility resulting in the transport of smaller mitochondria to other cellular compartments^44^. However, we had shown in that previous study that mitochondrial motility in MB neurons is not necessary for memory formation after massed training. Increased mitochondrial fusion is another process that could result in the observed change in mitochondria distribution. Thus, we shifted the mitochondrial fission–fusion equilibrium towards fusion by downregulating the expression of the main actor of mitochondrial fission, dynamin-related protein 1 (Drp1)^45^. Using the same approach as in Fig. 4a, we observed that KD of Drp1 in adult MB neurons resulted in an increase of the density of large mitochondria in the soma as compared with the genotypic controls (Fig. 4b). As elongated mitochondria have been associated with increased metabolic capacity, we investigated ATP production when Drp1 was knocked down in adult MB neurons. As in Figs. 1e and 2g, we measured ATP consumption in the soma of MB neurons of flies subjected to massed training. As previously observed, massed training increased ATP production in control flies. In Drp1 KD flies, ATP production was basally increased, at a level similar to that observed in control flies subjected to massed training (Fig. 4c and Extended Data Fig. 6b,c). Remarkably, expressing both Drp1 and MTPα RNAi in adult MB neurons abolished the increase of ATP production observed in Drp1 KD flies (Fig. 4d and Extended Data Fig. 6d), linking elevated ATP production in Drp1 KD MB neurons with FA β-oxidation. We then investigated whether this genetically induced enhancement of β-oxidation affects memory formation after massed training. Strikingly, downregulating Drp1 expression in the adult MB resulted in an improved memory performance after massed training (Fig. 4e, Extended Data Fig. 6e and Supplementary Table 6). In contrast, memory formation after spaced training was impaired in those flies (Extended Data Fig. 6e), as expected when mitochondrial motility is impaired by increased mitochondrial size^44,46,47^. These results were replicated with a second non-overlapping Drp1 RNAi line, as well as with an RNAi targeting Tango11, the homologue of human mitochondrial fission factor^48^ (Extended data Fig. 6f,g and Supplementary Table 6).

To investigate the mitochondrial metabolic pathway sustaining the improvement of memory performance after massed training in Drp1 KD flies, we co-expressed Drp1 RNAi with an RNAi targeting either FA mitochondrial metabolism (CPT1), pyruvate metabolism (PDH)^14^ or ketone body metabolism (ACAT1)^17^. Consistent with ATP imaging experiments, CPT1 KD was the only one of the three conditions that precluded the increase in memory performance induced by Drp1 KD (Fig. 4f,g and Extended Data Fig. 6h). Altogether, our data establish that favouring mitochondrial FA metabolic capacity (here by genetically impairing mitochondrial fission in MB neurons) improves memory performance after massed training. Thus, while the loss-of-function experiments presented earlier (Figs. 1–3) could be interpreted as supportive of neuronal activity within a classical framework of cellular energy metabolism, this result additionally shows that boosting energy production per se can be sufficient to improve cognitive performance.

Our study reveals an in vivo context, in a healthy brain, where FAs are used by neurons as an energy source to support memory formation. In humans, deficiency in the DDHD2 lipase is associated with cognitive impairment^49^, and in line with this, a constitutive knock out mouse model exhibits LTM deficits^50^. However, because FA metabolism is critical for other aspects of neuronal physiology, such as membrane turnover and protein acylation to regulate gene expression, it was difficult to conclude from these studies whether FAs generated by DDHD2-mediated lipolysis are used for energy production in neurons. While it was recently shown that DDHD2 is required in vitro for ATP production in neurons^11,12^, these studies suggest that FAs are coming from a neuronal LD internal store. Our study does not rule out the possibility of a limited internal store of FA as the presence of LDs in the MB has been shown by us and others^9,10^. However, we here show that, to support cognitive function, in vivo, FAs are synthesized in glia and provided to neurons as lipoproteins. In other learning paradigms (single learning session or spaced training), we previously showed that pyruvate-dependent memories are also fuelled by glycolysis-derived metabolites from cortex glia. Similarly, in food-deprived flies, cortex glia synthesize and export ketone bodies to allow memory formation by MB neurons. Together with our previous works^17,30,51^, a general framework thus emerges, in which glia provides a highly versatile metabolic support to MB neurons, enabling the proper formation of memory depending on the feeding status of the fly (starved versus fed) or the learning paradigm (cramming versus spaced practice). Further investigation will determine whether this dialogue between glia and neurons, which allows for metabolic plasticity, is specific to the associative memory centre or shared with other centres involved in complex behaviour.

Remarkably, it was recently shown in murine and human in vitro models that lipoprotein uptake in neurons via the sortilin lipoprotein receptor activates a transcriptional programme to favour neuronal use of FA as an energy substrate^52^. It suggests that neuronal metabolic plasticity could be triggered by specific signalling pathways. Whether such paths are activated in the context of memory formation will need further investigation.

Altogether, our data demonstrate that neuronal FA oxidation is required in MB neurons to sustain the formation of memory induced by cramming-like learning, thus uncovering a metabolic flexibility in neurons to support memory formation. In addition to providing new perspectives in the brain energy metabolism field, the improvement in memory performance after cramming-like learning by increasing mitochondrial metabolic capacity provides strong support for the concept of mitochondrial plasticity as a key factor of memory robustness^53^.

## Methods

### Fly strains

*Drosophila melanogaster* flies were raised on standard food medium (inactivated yeast 6% w/v, corn flour 6.66% w/v, agar 0.9% w/v, and methyl-4-hydroxybenzoate 22 mM) on a 12 h:12 h light–dark cycle at 18 °C and 60% humidity. The Canton Special (CS) strain was used as the wild-type strain. All lines were outcrossed for at least three generations to flies carrying a CS wild-type background. For behavioural experiments, both male and female flies were used. For in vivo imaging, immunostaining and LD-labelling experiments, female flies were used because of their larger size, as done previously^14,17,44,51^. Before imaging or behavioural experiments, we chose flies informally in a random manner from a much larger group raised together for all studies; there was no formal randomization procedure for selecting flies. As samples were allocated to experimental groups based on genotype, it was not possible to randomize the data collection process. Data collection and analysis were not performed blind to the conditions of the experiments. All strains used in this study are described in Supplementary Table 7. Pan-neuronal expression of transgenes was achieved using the elav-Gal4 line^54^. Pan-glial expression of transgenes was achieved using the Repo-Gal4 line^55^. For transgene expression in MB neurons we used the VT30559*-*Gal4 line^14^, and for transgene expression in cortex glia we used the R54H02-Gal4 driver^27^. In order to restrict UAS/GAL4-mediated expression to the adult stage, we used the TARGET system^21^ with the tub-Gal80^ts^ line to construct the inducible driver line tub-Gal80^ts^;VT30559-Gal4 and the tub-Gal80^ts^; R54H02-Gal4 previously described in refs. ^14^ and ^30^, respectively. Gal4 activity was released by transferring 1–2-day-old adult flies to 30 °C for 2–3 days. In some behavioural experiments (Extended Data Figs. 1d and 5b), the UAS-Dicer2 transgene was used in combination with the MB inducible driver (tub-Gal80^ts^;UAS-Dcr2, VT30559-Gal4) to increase either MTPα RNAi GD11299 or Bmm RNAi GD5139 efficiency, an approach that our laboratory successfully used in a previous study^17^.

For RNAi lines not yet validated, the efficiency of each RNAi construct to decrease the mRNA level of the targeted gene was confirmed following the protocol detailed in ‘Quantitative PCR analyses’ section, with results presented in Supplementary Table 2.

### Olfactory conditioning and memory test

The behaviour experiments, including sample sizes, were conducted similarly to previous studies from our research group^14,15,44^. For RNAi induction, 1–2-day-old flies were kept at 30 °C for 2–3 days until conditioning. The non-induced control flies, in which RNAi expression is inhibited, were kept at 18 °C. For all experiments, training and testing were performed at 25 °C and 80% humidity; after conditioning, flies were kept at 18 °C until testing. Briefly, groups of approximately 30–40 flies were subjected to one of the following olfactory conditioning protocols: five consecutive associative training cycles (5× massed) or five associative cycles spaced by 15-min inter-trial intervals (5× spaced). Custom-built barrels allowing parallel training of up to six groups were used for conditioning. Throughout the conditioning protocol, each barrel was plugged into a constant airflow at 2 l min^−1^. The sequence of one conditioning cycle consisted of an initial 90-s period of non-odorized airflow, followed by 60 s of the conditioned odour paired with 12 pulses of electric shocks (60 V; 1 pulse every 5 s with a pulse duration of 1.2 s). After 45 s of non-odorized airflow, the second odour was presented for 60 s without electric shocks, followed by 45 s of non-odorized airflow. The odorants 3-octanol (>95% purity; Fluka 74878, Sigma–Aldrich) and 4-methylcyclohexanol (99% purity; Fluka 66360) were diluted in paraffin oil at 0.360 mM and 0.325 mM, respectively, and were alternately used as conditioning stimuli. During unpaired conditionings, the odour and shock stimuli were delivered separately in time, with shocks starting 3 min before the first odorant.

The memory test was performed in a T-maze apparatus, typically after 24 h of massed or spaced training. Flies were exposed simultaneously to both odorants for 1 min in the dark. The performance index (PI) was calculated as the number of flies attracted to the unconditioned odour minus the number of flies attracted to the conditioned odour, divided by the total number of flies in the experiment; the resulting number was multiplied by 100. To avoid giving disproportionate statistical weight to a small number of flies, rare behavioural experiments involving a group of less than six flies were excluded. A single PI value is the average of two scores obtained from two groups of genotypically identical flies conditioned in two reciprocal experiments, using either odorant (3-octanol or 4-methylcyclohexanol) as the conditioning stimulus. The indicated ‘*n*’ is the number of independent PI values for each genotype.

Olfactory avoidance and shock avoidance tests were conducted similarly to previous studies from our research group^14,15,44^. The shock response tests were performed at 25 °C by placing flies in two connected compartments; electric shocks were administered in only one of the compartments. Flies were given 1 min to move freely in these compartments, after which they were trapped, collected and counted. The compartment in which the electric shocks were delivered was alternated between two consecutive groups. Shock avoidance was calculated as for the memory test. Since the delivery of electric shocks can modify olfactory acuity, our olfactory avoidance tests were performed on flies that had first been presented another odour paired with electric shocks. Innate odour avoidance was measured in a T-maze similar to those used for memory tests, in which one arm of the T-maze was connected to a bottle containing odour diluted in paraffin oil and the other arm was connected to a bottle with paraffin oil only. Naive flies were given the choice between the two arms during 1 min. The odour-interlaced side was alternated for successively tested groups. Odour concentrations used in this assay were the same as for the memory assays. At these concentrations, both odorants are innately repulsive.

### In vivo ATP imaging

As in all previous imaging studies from our laboratory^14,15,44^, in vivo imaging experiments were performed in female flies owing to their larger size, which facilitates surgery. Briefly, female flies carrying a tub-Gal80ts;VT30559-GAL4 construct were crossed to CS males or to males carrying either the UAS-AT1.03NL transgene, the UAS-AT1.03RK transgene or the appropriate UAS-RNAi together with the UAS-AT1.03NL transgene. Crosses for imaging experiments were raised at 23 °C to avoid RNAi expression during development. The 1–2-day-old adult progeny were induced for 3 days at 30 °C. After either the associative massed training or unpaired protocol, a single fly was affixed to a plastic coverslip using a non-toxic dental glue (Protemp II 3 M ESPE), and a 90 μl droplet of an artificial haemolymph solution was added on top of the coverslip. The composition of the artificial haemolymph solution was 130 mM NaCl (Sigma, S9625), 5 mM KCl (Sigma, P3911), 2 mM MgCl_2_ (Sigma, M9272), 2 mM CaCl_2_ (Sigma, C3881), 5 mM d-trehalose (Sigma, T9531), 30 mM sucrose (Sigma, S9378) and 5 mM HEPES-hemisodium salt (Sigma, H7637). Surgery was performed, as previously described^14,15,44^, to expose the brain for optical imaging. At the end of the surgery, a fresh 90-μl drop of the appropriate saline solution was applied on the aperture in the fly’s head. Two-photon imaging was performed on a Leica TCS-SP5 upright microscope equipped with a 25×, 0.95 NA water immersion objective. Two-photon excitation of cyan fluorescent protein (CFP) was achieved using a Mai Tai DeepSee laser tuned to 820 nm. Following this, 512 × 200 images were acquired at a frame rate of two images per second, and the entire duration of each recording was 360 s. After 2 min of baseline acquisition, 10 µl of a 50 mM sodium azide solution (Sigma, 71289; prepared in the same artificial haemolymph solution) was injected into the 90-μl droplet bathing the fly’s brain, bringing the sodium azide to a final concentration of 5 mM. To analyse the ATP imaging experiments, regions of interest (ROIs) were delimited by hand around each visible MB soma region, and the average intensity of the CFP and yellow fluorescent protein (YFP) channels over each ROI was calculated over time after background subtraction. The ATP sensor was designed so that FRET from CFP to YFP increases as the ATP concentration increases, and thus the FRET ratio was calculated as YFP intensity divided by CFP intensity. This ratio was normalized by a baseline value calculated over 120 s, starting at 120 s after the drug injection (corresponding to the last 120 s of the recording), when the ATP level was below the detection limit of the sensor. The slope was calculated between 90% and 30% of the plateau, which allowed the level of ATP consumption to be estimated, using the same principle as in ref. ^14^ to measure MB pyruvate consumption. Imaging analysis was performed using a custom-written MATLAB script^14^. The indicated ‘*n*’ is the number of animals assayed in each condition.

### Immunostaining, image acquisition and analysis for STED microscopy

The immunostaining for STED experiments, as well as the image acquisitions and analysis, were conducted similarly to our previous study^44^. Flies were raised at 18 °C; to induce RNAi expression as well as mtDsRed, adult flies were kept at 30.5 °C for 3 days before conditioning with either the 5× massed associative training or unpaired protocol. One hour after the end of the training period, whole adult flies were fixed in 4% paraformaldehyde (Electron Microscopy Sciences) in phosphate-buffered saline containing 0.6% Triton X-100 (PBST) at 4 °C overnight. Next, brains were dissected in PBS solution and fixed again for 1 h at room temperature (RT) in 4% paraformaldehyde in PBST followed by three 20-min rinses in PBST, with blocking for 2 h at RT with 2% bovine serum albumin (BSA; Sigma–Aldrich, A9085) in PBST. Samples were incubated with the primary antibody Atto647N FluoTag-X4 anti-RFP (NanoTag, N0404-Atto647N-L) at 1:100 in the blocking buffer at 4 °C overnight. The next day, brains were rinsed twice for 20 min in PBST and once for 20 min in PBS. Samples were then mounted in ProLong Gold Antifade Mountant (Invitrogen) using precision cover glasses (thickness: no. 1.5H; Marienfeld Superior).

Single-colour 3D-STED imaging was performed using a Leica TCS SP8 STED 3X microscope (excitation at 633 nm, STED depletion laser at 775 nm (pulsed), with 60% in *x* and *y* dimensions and 50% in the *z* dimension) with a 93× motCorr glycerol immersion objective (NA of 1.3). Images of one MB soma region per brain were obtained with a voxel size of 41.68 nm (*x*) × 41.68 nm (*y*) × 72.48 nm (*z*). Note that the MB lobes are situated too deep within the brain (70–120 µm as compared with 10–30 µm for the MB somas) to be imaged using 3D-STED microscopy.

One ROI per brain, containing only somas, was delimited from the full image (120 µm × 120 µm × 8 µm) using a constant square box of 21 µm × 21 µm × 8 µm. The raw ROIs were then analysed using Icy Software and the HK-mean plugin followed by the connected-component (ROI extraction mode and removal of the border objects) plugin to detect mitochondria and their volumes^56,57^. Each ROI typically contained 200–350 mitochondria. The same parameters (that is, the Gaussian pre-filter and the intensity classes) were used for all ROIs analysed in the study. These parameters were first determined on a set of ROIs from brain samples by a researcher blinded to the genotype and the behavioural training. The volume of the minimal envelope containing all of the detected mitochondria in each ROI was determined using a custom-written ImageJ macro^58^. Briefly, we proceeded with the binarization of single fluorescent images of the *z-*stack series obtained from the Icy analysis. Binarized fluorescent objects were then connected using XOR and Convex Hull mathematical operators. Surface and volume estimates were performed using the 3D object option available in Fiji.

On the basis of the mitochondria volume distribution in the control group (5× massed unpaired, tub-Gal80^ts^>UAS-mtDsRed), we defined four categories of mitochondria, from the smallest volume to the largest, using the mean of the 25th and 75th percentiles and the medians of mitochondria volumes in each ROI from one fly brain (Extended Data Fig. 3a). The limit of each category was defined as follows: for category 1 (the smallest), the upper limit is the mean of the 25th percentiles; for category 2, the lower limit is the mean of the 25th percentiles and the upper limit is the mean of the medians; for category 3, the lower limit is the mean of the medians and the upper limit is the mean of the 75th percentiles; and for category 4 (the largest), the lower limit is the mean of the 75th percentiles. The same volume limits were used to define the categories in all experimental conditions. To allow comparisons between the samples (for each ROI from one fly brain), we normalized the number of mitochondria in each category by the volume of the minimal envelope containing all of the detected mitochondria to obtain the mitochondria density for each category.

### LD imaging and image analysis

To achieve LD imaging in MB neurons, VT30559-GAL4 flies were crossed with flies bearing UAS-LD-GFP, with or without the appropriate UAS-RNAi, and raised at 25 °C. Then, 1–3-day-old whole adult flies were fixed in 4% paraformaldehyde (Electron Microscopy Sciences) in PBST at 4 °C overnight. Next, brains were dissected in a PBS solution and fixed again for 1 h at RT in 4% paraformaldehyde in PBST. Brains were then rinsed once in PBST for 20 min, and twice in PBS for 20 min. After rinsing, brains were mounted using Prolong Mounting Medium (Invitrogen). Images of one MB soma region per brain were acquired the following day with a voxel size of 0.117 µm (*x*) × 0.117 µm (*y*) × 0.41 µm (*z*) on an Olympus FV1000 confocal microscope with a 60×, 1.35 oil-immersion objective using a 473-nm laser. Confocal *z* stacks were imported into Fiji^59^ and CellProfiler 4.2.6 software^60^ for further analyses. Using Fiji, one ROI per brain, containing only MB somas, was delimited from the full image using a constant rectangular box of 120 µm × 90 µm × 2 µm. Next, CellProfiler was used for 3D object detection and counting to identify LDs from the background staining of the plasma membrane. Thresholding was automatically performed on the basis of the Robust Background thresholding method. This method assumes that the background distribution approximates a Gaussian distribution as we previously carried out for the LD analysis^17^. Specifically, the threshold background parameters used for the lower and upper outlier fractions were fixed to 0.02 and 0.01, respectively, with a mean averaging method, and a variance method with the standard deviation set to 4 and a threshold correction factor of 5. The watershed module was used to detect the 3D objects, and a filter was applied on the basis of the object mean intensity to discard residual background from non-specific labelling of the plasma membrane (a minimum intensity value of 0.025). The number and volume of LDs were obtained using the ‘measure object intensity’ and ‘measure object size’ modules. To allow comparisons between the samples, we normalized the number of LDs by the volume of the MB soma region. This volume was estimated on the basis of the background signal from the plasma membrane. Here, the object was defined using manual thresholding that set the lower limit to 0.00005, image smoothing with a sigma of 1 and applying a median filter (window of 10). Then, the ‘removeholes’ module, with the ‘measure image area occupied’ module, was used to measure the volume occupied by the MB soma region. The indicated ‘*n*’ is the number of animals that were assayed in each condition.

### LD staining with BODIPY

#### Neutral LD

Neutral LDs were stained with the non-polar fluorescent probe BODIPY^493/503^ (Sigma, D3922) and analysed as previously described^17^. Female flies carrying the tub-Gal80^ts^;R54H02 cortex glia-specific driver were crossed with males carrying the specified UAS-RNAi or with CS males. Flies were reared at 18 °C, and 1–2-day-old adults were shifted to 30 °C for 2 days. Brains were dissected on ice in 1× PBS (Sigma, P4417), fixed for 30 min in 4% paraformaldehyde (Electron Microscopy Sciences, 15710), washed three times with 1x PBS and incubated for 30 min in the dark with 1 µM BODIPY^493/503^ (Sigma, D3922). After three PBS washes, samples were mounted in ProLong (Life Technologies, P36965) and imaged the same day on a Nikon A1R confocal (100×, 1.40 NA oil objective; 488-nm excitation; 1,024 × 1,024), acquiring *z* stacks in cortex glia near the MB calyx. Images were analysed in Fiji (ImageJ 1.52p)^59^ and CellProfiler Analyst 3.1.9 software^61^. From each stack, a single plane was converted to 8-bit and an 84µm × 80 µm ROI adjacent to the calyx was selected. To remove the plasma membrane BODIPY signal, the ROI intensity histogram was Gaussian fitted in Prism 8.0, excluding *x* < 3 and *x* > 40; a global LD threshold of (mean + 4 × s.d.)/255 was applied to all images. Objects were counted as LDs if their area was 0.37–1.5 µm² on the basis of previous LD data in literature^62,63^. For each ROI, individual LD areas (square micrometre) were measured to compute mean LD area. One ROI per hemisphere was analysed and averaged per brain; if only one hemisphere was scorable, a single ROI was used. The indicated ‘*n*’ corresponds to the number of brains analysed.

#### Peroxidized lipid staining

LDs were stained with the fluorescent lipid peroxidation sensor C11-BODIPY^581/591^ (Invitrogen, D3861), as previously described^28^. Briefly, 1–2-day-old adults female CS flies, subjected to either associative massed training or the unpaired protocol, were cold-anaesthetised immediately after conditioning. Their brains were dissected on ice in 1× PBS (Sigma, P4417), incubated for 30 min in the dark with 20 µM C11-BODIPY^581/591^ in a 1% BSA and 1× PBS solution. The C11-BODIPY^581/591^ stock solution was 39.7 mM in dimethylsulfoxide (DMSO; BioUltra, 41639). The brains were washed once with 1× PBS, mounted in ProLong Gold mounting medium (Invitrogen, P36930) and images were obtained on the same day. Then, 1,024 × 1,024 images were acquired with a Nikon A1R confocal microscope (100×, 1.40 NA oil-immersion objective lens), with *z* stacks in the cortex glia near the MB calyx, with alternate excitation to detect the non-oxidized (excitation of 561 nm and emission of 570–610 nm) and oxidized (excitation of 488 nm and emission of 500–540 nm) forms. From each stack, a single plane adjacent and atop to the MB calyx was selected and a 60 µm × 60 µm ROI was selected using Fiji (ImageJ 1.52p)^59^ to measure the mean ROI intensity in each channel. The oxidation level was calculated as the ratio between the ROI mean intensity values of the green (oxidized) channel and the red (non-oxidized) channel.

### Immunohistochemistry experiments

Female flies carrying the Mi{Trojan-GAL4.1}bmm^MI13321-TG4.1^ construct^64^ were crossed to male flies carrying the UAS-mCD8::GFP construct^65^. Before dissection, 2–4-day-old female flies were fixed in 4% paraformaldehyde in PBST at 4 °C overnight. Fly brains were dissected on ice in PBS solution and rinsed three times for 20 min in PBST. Then, brains were blocked with 2% BSA in PBST for 2 h. Next, samples were incubated with primary antibodies in the blocking solution (2% BSA in PBST) at 4 °C overnight. The following primary antibodies were used: 1:250 rabbit anti-GFP (Invitrogen, A11122) and 1:100 mouse anti-nc82 (DSHB, nc82). The following day, brains were rinsed three times for 20 min with PBST and then incubated for 3 h at RT with secondary antibodies diluted in blocking solution. The following secondary antibodies were used: 1:400 anti-rabbit conjugated to Alexa Fluor 488 (Invitrogen, A11034) and 1:400 anti-mouse conjugated to Alexa Fluor 594 (Invitrogen, A11005). Brains were then rinsed once in PBST for 20 min and twice in PBS for 20 min. After rinsing, brains were mounted using Prolong Mounting Medium (Invitrogen). Acquisitions were made with a Nikon A1R confocal microscope with a 40×, 1.15 water immersion objective.

### Quantitative PCR analyses

To assess the efficiency of each RNAi line used in this study, quantitative PCR analyses were conducted similarly to previous studies from our research group^17,44^. Female flies carrying the appropriate driver (elav-Gal4, Repo-Gal4 pan-glial or VT30559-Gal4) were crossed with males carrying the specified UAS-RNAi or with CS males. Fly progeny was reared at 25 °C throughout their development. Then, 1–2-day-old flies were transferred to fresh food for 1 day prior to RNA extraction. RNA extraction, DNAse I treatment (when needed) and cDNA synthesis were performed as previously described^17^ using the RNeasy Plant Mini Kit (Qiagen), RNA MiniElute Cleanup Kit (Qiagen), DNAse I treatment (BioLabs), oligo(dT)20 primers and the SuperScript III First-Strand kit (Thermo Fisher Invitrogen). Amplification was performed using a LightCycler 480 (Roche) and the SYBR Green I Master mix (Roche). Specific primers used for each gene cDNA and the reference α-Tub84B (Tub, CG1913) cDNA are provided in Supplementary Table 8. Reactions were performed in triplicate. The specificity and size of amplification products were assessed by melting curve analyses. The level of cDNA for each gene of interest was compared against the level of the α-Tub84B reference cDNA. Expression relative to the reference was expressed as a ratio (2^−ΔCp^, where Cp is the crossing point).

### Statistical analysis

Data are expressed as the mean ± s.e.m. with dots as individual values (one experimental replicate, *n* = 1) corresponding to: a group of 40–50 flies analysed together in a behavioural assay; the response of a single recorded fly for ATP imaging; one brain for LD experiments or for mitochondrial morphology analysis; or one mRNA extraction from heads of a group of 50 flies used for a quantitative PCR experiment. Statistical analysis was performed using the GraphPad Prism 8.0 software (GraphPad Software). No statistical methods were used to pre-determine sample sizes but our sample sizes are similar to those reported in previous publications^14,17,44^. Data distribution was assumed to be normal but this was not formally tested. Comparisons between two groups were performed by unpaired two-sided Student’s *t*-test, with results given as the value *t*_*x*_ of the *t* distribution, where *x* is the number of degrees of freedom. Comparisons between more than two groups were performed by one-way analysis of variance (ANOVA) with post hoc testing by the Newman–Keuls pairwise comparisons test between the experimental group and its controls (significance denoted by *P* < 0.05). ANOVA results are given as the value of the Fisher distribution *F*_(*x,y*)_, where *x* is the number of degrees of freedom numerator and *y* is the total number of degrees of freedom denominator. Asterisks in each figure refer to the post hoc comparison between the genotype of interest and the genotypic controls. The nomenclature used corresponds to *P < 0.05, **P < 0.01, ***P < 0.001 and ****P < 0.0001; NS denotes not significant, where *P* > 0.05. Figures were made using Affinity Designer v2.

### Reporting summary

Further information on research design is available in the Nature Portfolio Reporting Summary linked to this article.

## Supplementary information

Supplementary Information Supplementary Tables 1–8.

Reporting Summary

## Source data

Source Data Fig. 1 Numerical source data.

Source Data Fig. 2 Numerical source data.

Source Data Fig. 3 Numerical source data.

Source Data Fig. 4 Numerical source data.

Source Data Extended Data Fig. 1 Numerical source data.

Source Data Extended Data Fig. 2 Numerical source data.

Source Data Extended Data Fig. 3 Numerical source data.

Source Data Extended Data Fig. 4 Numerical source data.

Source Data Extended Data Fig. 5 Numerical source data.

Source Data Extended Data Fig. 6 Numerical source data.

## Data Availability

No datasets that require mandatory deposition into a public database were generated during the current study. Processed data from imaging experiments and raw data of behavioural assays are provided as Source data files alongside the publication. Unprocessed images, which represent a large volume, will be available through e-mailing the corresponding authors, and will be shared without restriction. Source data are provided with this paper.

## Code availability

No new code was generated during this study, which involved codes previously reported in refs. ^14,44,58^.
