# Supplementary material for: Neuronal fatty acid oxidation fuels memory after intensive learning in Drosophila
Source: Nat Metab. 2025 Dec 10;7(12):2438–50. doi: 10.1038/s42255-025-01416-5 (PMC12727536; doi:10.1038/s42255-025-01416-5)
Supplement: Supplementary file 1 — Supplementary Tables 1–8. [file 42255_2025_1416_MOESM1_ESM.pdf]

nature metabolism

Supplementary information https://doi.org/10.1038/s42255-025-01416-5

Neuronal fatty acid oxidation fuels memory after intensive learning in *Drosophila*

In the format provided by the authors and unedited

Control experiments for olfactory acuity and electric shock avoidance.

Expression of the different RNAi constructs used in this study in MB neurons or in Cortex glia did not have any significant effect on olfactory acuity or the avoidance of electric shocks. The *P*-value indicated for RNAi-expressing flies is the lowest one obtained from the two pairwise comparisons between these flies and their driver (tubulin-GAL80ts; VT30559/+ or tubulin-GAL80ts; R54H02) or effector (UAS-…RNAi/+) controls. n is indicated for each experiment.

Supplementary Table 1: Control experiments for olfactory acuity and electric shock avoidance related to Fig. 1 and Extended Data Fig. 1.

Genotype	Shock avoidance		Naive odor avoidance			
			Octanol		Methylcyclohexanol	
	Mean ± s.e.m.	Statistics	Mean ± s.e.m.	Statistics	Mean ± s.e.m.	Statistics
tubGal80^{ts};VT30559/+	54.8±9.7	$F_{2,21}$=0.51<br>p=0.61<br>n=8	70.6±4.4	$F_{2,21}$=0.027<br>p=0.97<br>n=8	55.4±2.2	$F_{2,21}$=1.04<br>p=0.37<br>n=8
tubGal80^{ts};VT30559><br>UAS-CPT1 RNAi HMS0040	63.8±11.4		68.8±6.1		67.5±7.8	
+/UAS-CPT1 RNAi HMS0040	49.3±9.7		69.4±6.8		66.9±8.3	
tubGal80^{ts};VT30559/+	70.1±3.9	$F_{2,45}$=0.35<br>p=0.71<br>n=16	48.9±4.6	$F_{2,33}$=0.74<br>p=0.49<br>n=12	46.6±6.4	$F_{2,33}$=0.14<br>p=0.87<br>n=12
tubGal80^{ts};VT30559><br>UAS-CPT1 RNAi KK100935	72.1±4.0		46.8±4.5		50.4±5.8	
+/UAS-CPT1 RNAi KK100935	67.3±4.2		42.1±2.9		47.3±3.3	
tubGal80^{ts};VT30559/+	43.4±7.3	$F_{2,21}$=0.53<br>p=0.60<br>n=8	41.1±6.6	$F_{2,21}$=0.28<br>p=0.76<br>n=8	40.4±7.6	$F_{2,21}$=0.11<br>p=0.89<br>n=8
tubGal80^{ts}; VT30559><br>UAS-MTPα RNAi HMS00660	36.1±12.5		34.8±7.3		35.4±4.9	
+/UAS-MTPα RNAi HMS00660	30.0±6.7		35.3±6.1		38.1±9.2	
tubGal80^{ts}; UAS-Dcr2, VT30559/+	70.2±5.7	$F_{2,27}$=0.36<br>p=0.70<br>n=10	56.4±3.9	$F_{2,39}$=1.78<br>p=0.18<br>n=14	56.8±4.8	$F_{2,39}$=0.21<br>p=0.81<br>n=14
tubGal80^{ts}; UAS-Dcr2, VT30559><br>UAS-MTPα RNAi GD11299	65.5±3.6		50.1±3.2		53.1±3.9	
+/UAS-MTPα RNAi GD11299	71.0±5.3		47.7±2.8		53.1±5.1	
tubGal80^{ts};VT30559/+	51.3±3.8	$F_{2,27}$=0.28<br>p=0.76<br>n=10	59.7±11.4	$F_{2,24}$=0.86<br>p=0.44<br>n=9	77.1±9.4	$F_{2,24}$=0.18<br>p=0.84<br>n=9
tubGal80^{ts}; VT30559><br>UAS-HAD1 RNAi HMC05280	51.1±9.6		40.4±10.9		70.2±7.5	
+/UAS-HAD1 RNAi HMC05280	57.5±6.0		45.0±10.2		76.4±9.7	
+/yw	41.0±10.9	t_{14}=0.78<br>p=0.45<br>n=8	62.4±7.3	t_{14}=0.41<br>p=0.69<br>n=8	53.9±10.9	t_{14}=1.60<br>p=0.13<br>n=8
+/HAD1^{nl}	51.3±7.5		66.3±6.1		74.9±7.2	

Supplementary Table 2: Efficiency of genetic knockdowns used in the study.

Statistical comparisons were made using a two-sided unpaired t-test. Asterisks illustrate the significance level, with the following nomenclature: ***P<0.001, **P<0.01; *P<0.05.

Genotype	Mean ± s.e.m.	Statistics	% of mRNA reduction
elav/+	1.05±0.07	t_4=7.461 p=0.0017 (**) n=3	79%
elav>UAS-CPT1 RNAi HMS0040	0.23±0.09		
elav/+	0.77±0.67	t_4=4.150 p=0.0060 (**) n=4	60%
elav>UAS-CPT1 RNAi KK100935	0.31±0.09		
elav/+	0.90±0.27	t_6=2.707 p=0.0353 (*) n=4	80%
elav>UAS-MTPα RNAi HMS00660	0.18±0.01		
elav/+	1.02±0.26	t_4=3.256 p=0.0312 (*) n=3	84%
elav>UAS-MTPα RNAi GD11299	0.16±0.03		
elav/+	1.09±0.07	t_4=5.626 p=0.0049 (**) n=3	75%
elav>UAS-HAD1 RNAi HMC05280	0.27±0.12		
+/yw	1.19±0.12	t_4=3.578 p=0.0232 (*) n=3	50%
+/$Had1^{nl}$	0.59±0.11		
Repo/+	0.017±0.006	t_{12}=2.594 p=0.024(*) n=7	95%
Repo>UAS-ACC RNAi GD3482	0.001±0.0003		
Repo/+	0.043±0.003	t_4=6.292 p=0.0033 (**) n=4	77%
Repo>UAS- Apoltp RNAi HMC03294	0.010±0.004		
elav/+	1.476±0.341	t_4=3.186 p=0.033 (*) n=4	78%
elav>UAS- Lrp1 RNAi HMS02875	0.316±0.127		
elav/+	2.09±0.25	t_6=4.365 p=0.047 (**) n=4	57%
elav>UAS-FABP RNAi HMS01163	0.89±0.10		
elav/+	2.33±0.21	t_6=7.597 p=0.0003 (***) n=4	75%
elav>UAS-FABP RNAi KK116001	0.59±0.10		
elav/+	0.08±0.01	t_5=3.281	

elav>UAS-Drp1 RNAi HMC03230	0.05±0.00	p=0.0219 (*)<br>n=3-4	38%
elav/+	0.08±0.01	t_5=2.699<br>p=0.0428 (*)<br>n=3-4	38%
elav>UAS-Drp1 RNAi GD10456	0.05±0.01		

Supplementary Table 3: Efficiency of genetic knockdowns targeting MB neurons.

Statistical comparisons were made using a two-sided unpaired t-test. Asterisks illustrate the significance level, with the following nomenclature: *P<0.05.

Genotype	Mean ± s.e.m.	Statistics	% of mRNA reduction
VT30559/+	0.33±0.01	t_4=3.118 p=0.035 (*) n=3	17%
VT30559>UAS-CPT1 RNAi KK100935	0.28±0.02		
VT30559/+	0.47±0.03	t_6=2.398 p=0.0535 n=4	25%
VT30559>UAS-MTPα RNAi GD11299	0.36±0.03		
VT30559/+	0.015±0.006	t_9=2.291 p=0.048 (*) n=5-6	85%
VT30559>UAS-HAD1 RNAi HMC05280	0.003±0.0006		
VT30559/+	0.36±0.05	t_{12}=2.482 p=0.028 (*) n=7	35%
VT30559>UAS-Bmm RNAi JF09146	0.24±0.02		
VT30559/+	7.78±0.54	t_{10}=2.389 p=0.038 (*) n=6	23%
VT30559>UAS-FABP RNAi HMS01163	5.98±0.52		

Supplementary Table 4: Control experiments for olfactory acuity and electric shock avoidance related to Fig. 2 and Extended Data Fig. 4

Genotype	Shock avoidance		Naive odor avoidance			
			Octanol		Methylcyclohexanol	
	Mean ± s.e.m.	Statistics	Mean ± s.e.m.	Statistics	Mean ± s.e.m.	Statistics
tubGal80^{ts}; R54H02/+	26.9±6.7	$F_{2,21}$=0.89 p=0.43 n=8	30.0±6.3	$F_{2,21}$=0.23 p=0.79 n=8	49.8 ±7.1	$F_{2,21}$=1.37 p=0.27 n=8
tubGal80^{ts}; R54H02> UAS-ACC RNAi GD3482	37.7±5.2		34.6±5.9		41.0±7.9	
+/UAS-ACC RNAi GD3482	32.4±5.32		34.4±3.5		45.9±8.2	
tubGal80^{ts}; R54H02/+	42.1±7.5	$F_{2,21}$=1.70 p=0.21 n=8	35.1±6.2	$F_{2,21}$=0.87 p=0.43 n=8	51.9±6.9	$F_{2,21}$=0.38 p=0.69 n=8
tubGal80^{ts}; R54H02> UAS- Apolpp RNAi HM05157	35.4±7.5		34.9±5.6		45.6±2.8	
+/UAS- Apolpp RNAi HM05157	54.9±7.7		46.8±9.4		52.9±6.9	
tubGal80^{ts}; R54H02/+	50.4±10.0	$F_{2,21}$=0.33 p=0.72 n=8	43.3±6.9	$F_{2,21}$=1.1 p=0.36 n=8	43.9±7.8	$F_{2,21}$=0.31 p=0.73 n=8
tubGal80^{ts}; R54H02> UAS-Apoltp RNAi HMC03294	42.6±9.7		48.8±6.1		50.9±8.5	
+/UAS-Apoltp RNAi HMC03294	40.6±6.7		36.3±4.8		53.0±9.2	
tubGal80^{ts}; VT30559/+	43.5±8.3	$F_{2,21}$=0.20 p=0.82 n=8	36.9±6.2	$F_{2,21}$=0.54 p=0.59 n=8	37.1±8.8	$F_{2,21}$=0.92 p=0.41 n=8
tubGal80^{ts}; VT30559> UAS-Lrp1 RNAi HMS02875	40.4±9.4		46.4±8.3		51.6±8.3	
+/UAS-Lrp1 RNAi HMS02875	47.8±7.1		37.9±6.7		41.3±5.9	

Supplementary Table 5: Control experiments for olfactory acuity and electric shock avoidance related to Fig. 3 and Extended Data Fig. 5

Genotype	Shock avoidance		Naive odor avoidance			
			Octanol		Methylcyclohexanol	
	Mean ± s.e.m.	Statistics	Mean ± s.e.m.	Statistics	Mean ± s.e.m.	Statistics
tubGal80^{ts};VT30559/+	52.1±4.8	$F_{2,45}$=1.70 p=0.19 n=16	50.8±5.6	$F_{2,39}$=1.41 p=0.26 n=14	38.9±3.4	$F_{2,39}$=1.37 p=0.27 n=14
tubGal80^{ts};VT30559> UAS-Bmm RNAi JF09146	61.6±5.3		65.4±6.9		46.9±5.4	
+/UAS-Bmm RNAi JF09146	49.7±4.4		57.0±5.8		37.3±4.0	
tubGal80^{ts}; UAS-Dcr2, VT30559/+	36.8±6.2	$F_{2,33}$=0.11 p=0.90 n=12	76.3±4.1	$F_{2,33}$=1.9 p=0.17 n=12	72.5±5.4	$F_{2,29}$=1.22 p=0.31 n=10-11
tubGal80^{ts}; UAS-Dcr2, VT30559> UAS-Bmm RNAi GD5139	38.8±4.9		64.5±4.9		63.6±5.4	
+/UAS-Bmm RNAi GD5139	41.5±9.5		74.1±4.7		74.0±4.4	
tubGal80^{ts};VT30559/+	35.3±7.8	$F_{2,21}$=1.06 p=0.36 n=8	38.3±5.8	$F_{2,21}$=0.08 p=0.92 n=8	32.0±9.7	$F_{2,21}$=0.19 p=0.83 n=8
tubGal80^{ts};VT30559> UAS-FABP RNAi HMS01163	53.1±12.6		39.9±5.9		34.6±9.3	
+/UAS-FABP RNAi HMS01163	47.8±5.5		41.4±4.1		39.3±5.9	
tubGal80^{ts};VT30559/+	64.9±2.5	$F_{2,21}$=0.44 p=0.65 n=8	47.4±6.1	$F_{2,33}$=0.73 p=0.49 n=12	43.2±4.8	$F_{2,33}$=0.19 p=0.83 n=12
tubGal80^{ts};VT30559> UAS-FABP RNAi KK116001	61.9±4.6		56.1±6.7		45.1±4.5	
+/UAS-FABP RNAi KK116001/	59.9±4.0		47.3±4.9		47.7±6.2	

Supplementary Table 6: Control experiments for olfactory acuity and electric shock avoidance related to Fig. 4 and Extended Data Fig. 6.

Genotype	Shock avoidance		Naive odor avoidance			
			Octanol		Methylcyclohexanol	
	Mean ± s.e.m.	Statistics	Mean ± s.e.m.	Statistics	Mean ± s.e.m.	Statistics
tubGal80^{ts};VT30559/+	42.6±8.2	$F_{2,45}$=0.61 p=0.55 n=16	57.0±6.1	$F_{2,45}$=0.64 p=0.53 n=16	52.0±6.6	$F_{2,45}$=0.94 p=0.40 n=16
tubGal80^{ts};VT30559> UAS-Drp1 RNAi HMC03230	50.9±6.4		61.3±5.4		59.8±5.2	
+/UAS-Drp1 RNAi HMC03230	39.4±7.9		65.9±5.2		63.6±6.4	
tubGal80^{ts};VT30559/+	41.0±5.8	$F_{2,69}$=0.49 p=0.61 n=24	49.6±5.2	$F_{2,2}$=0.19 p=0.83 n=21-22	58.8±7.4	$F_{2,45}$=0.70 p=0.50 n=16
tubGal80^{ts};VT30559> UAS-Drp1 RNAi GD10456	48.3±8.3		46.1±6.2		60.3±5.3	
+/UAS-Drp1 RNAi GD10456	50.3±6.4		51.6±7.8		68.0±4.5	
tubGal80^{ts};VT30559/+	47.4±2.6	$F_{2,45}$=3.16 p=0.05 n=16	59.0±5.5	$F_{2,2}$=0.15 p=0.85 n=16	55.9±4.7	$F_{2,2}$=0.005 p=0.99 n=16
tubGal80^{ts};VT30559> UAS-Tango11 RNAi HMJ30309	58.4±4.1		59.4±4.4		55.8±5.2	
+/UAS-Tango11 RNAi HMJ30309	61.3±5.2		62.8±5.8		56.4±4.4	

Supplementary Table 7: ***Drosophila melanogaster*** **strains used in the study.**

STRAIN	SOURCE	IDENTIFIER
VT30559-Gal4	Vienna *Drosophila* Resource Center (VDRC)	v206077
tub-Gal80^{ts}	Bloomington *Drosophila* Stock Center (BDSC)	7019
tub-Gal80^{ts}; VT30559-Gal4	Plaçais et al. [14]	N/A
elav-Gal4	Luo et al. [54]	N/A
Repo-Gal4	Sepp et al.[55]	N/A
R54H02-Gal4	Kremer et al. [27]	N/A
tub-GAL80^{ts}; R54H02-Gal4	de Tredern et al. [30]	N/A
UAS-Dicer2	BDSC	24650
Tub-Gal80^{ts}; UAS-Dcr2, VT30559-Gal4	This study	N/A
UAS-GLaz RNAi KK106377	VDRC [29]	v107433
UAS-ACC RNAi GD3482	VDRC	v8105
UAS-Bmm RNAi GD5139	VDRC	v37877
UAS-FABP RNAi KK116001	VDRC	v109169
UAS-CPT1 RNAi KK100935	VDRC	v105400
UAS-MTPα RNAi GD11299	VDRC	v21845
UAS-Drp1 RNAi GD10456	VDRC	v44155
UAS-PDH RNAi KK107865	VDRC	v104022
UAS-ACAT1 RNAi GD7132	VDRC	v16099
UAS-GLaz RNAi HMC06329	BDSC	67228
UAS-Tango11 RNAi HMJ30309	BDSC	63996
UAS-Apolpp RNAi HM05157	BDSC	28946
UAS-Apoltp RNAi HMC03294	BDSC	51937
UAS-Lrp1 RNAi HMS02875	BDSC	44579
UAS-Bmm RNAi JF01946	BDSC	25926
UAS-FABP RNAi HMS01163	BDSC	34685
UAS-CPT1 RNAi HMS00040	BDSC	34066
UAS-MTPα RNAi HMS00660	BDSC	32873
UAS-HAD1 RNAi HMC05280	BDSC	62273
Had1^{nl}	BDSC	1037
UAS-Drp1 RNAi HMC03230	BDSC	51483
UAS-AT1.03NL	H. Imamura[25]	
UAS-AT1.03RK	H. Imamura[25]	
UAS-mtDsRed	BDSC	93056
Mi{Trojan-GAL4.1}bmm$^{MI13321\text{-}TG4.1}$	BDSC	67510
UAS-mcD8::GFP	Scheunemann et al. [65]	
UAS-LD-GFP	M. A. Welte[33]	
UAS-LD-GFP, UAS-Bmm RNAi JF01946	This study	N/A
UAS-LD-GFP, UAS-CPT1 RNAi HMS00040	This study	N/A
UAS-LD-GFP, UAS-MTPα RNAi HMS00660	This study	N/A
UAS-AT1.03NL; UAS-MTPα RNAi HMS00660	This study	N/A
UAS-AT1.03NL, UAS-Drp1 RNAi HMC03230	This study	N/A
UAS-mtDsRed, UAS-Drp1 RNAi HMC03230	This study	N/A
UAS-AT1.03NL, UAS-Drp1 RNAi HMC03230; UAS-MTPα RNAi HMS00660	This study	N/A
UAS-CPT1 RNAi KK100935; UAS-Drp1 RNAi GD10456	This study	N/A

UAS-PDH RNAi KK107865; UAS-Drp1 RNAi GD10456	This study	N/A
UAS-ACAT1 RNAi GD7132; UAS-Drp1 RNAi GD10456	This study	N/A

Supplementary Table 8: Primer sequences used for quantitative PCR.

Gene	Related to Figure:	Forward primer	Reverse primer
α-Tub84B	Fig. 1 and Extended Data Fig. 1	TTGTCGCGTGTGAAACACTTC	CTGGACACCAGCCTGACCAAC
CPT1	Fig. 1 and Extended Data Fig. 1	ACCATTGAGTCATCCGCCTG	CATCCTGGTAGCCCAACTGG
MTPα	Fig. 1 and Extended Data Fig. 1	CAGGAACCTCCAAGGACACC	TTCCTGCAGCAGTCTGATGG
HAD1	Fig. 1 and Extended Data Fig. 1<br>HAD1 RNAi HMC05280	AATGGGACCGACACCGAAAA	CTCAACTGGGTGAGGCAGTT
	Extended Data Fig. 1<br>$Had1^{nl}$ mutant	CAGGTGGTGCTGTACGACAT	GAGATGCAGGCGAATTGCTG
ACC	Fig. 2	ATGAGCGAAACAAATGAGTC CA	GGAACTCGTCACATGCCTCG
Apoltp	Fig. 2	ACGTTCACGGACACATCGAA	TCCGTCTCCAACTGAATGCC
Lrp1	Fig. 2	ATGGCGGTATTTGCAGGGAA	TTCTTGCAAAAATCGGCGCA
Bmm	Fig. 3 and Extended Data Fig. 5	CACTTCGATGCCACAGGAC	GACATCGTCCTCTAGCGGTG
FABP	Fig. 3 and Extended Data Fig. 5	TCAACAGTGAACATCTTGTGC	GCTGTTGCCCATCTTGCG
Drp1	Fig. 4 and Extended Data Fig. 6	ATCTACAGCCCACTCGATGAT	GAAGCACTTCTTGGTGTGCAG
