# Supplementary material for: Neuronal fatty acid oxidation fuels memory after intensive learning in Drosophila
Source: Nat Metab. 2025 Dec 10;7(12):2438–50. doi: 10.1038/s42255-025-01416-5 (PMC12727536; doi:10.1038/s42255-025-01416-5)
Supplement: Supplementary file 2 — Reporting Summary [file 42255_2025_1416_MOESM2_ESM.pdf]

nature portfolio

Corresponding author(s): Thomas Preat, Pierre-Yves Plaçais

Last updated by author(s): Oct 22, 2025

Reporting Summary

Nature Portfolio wishes to improve the reproducibility of the work that we publish. This form provides structure for consistency and transparency in reporting. For further information on Nature Portfolio policies, see our Editorial Policies and the Editorial Policy Checklist.

Statistics

For all statistical analyses, confirm that the following items are present in the figure legend, table legend, main text, or Methods section.

n/a	Confirmed	
☐	☒	The exact sample size (*n*) for each experimental group/condition, given as a discrete number and unit of measurement
☐	☒	A statement on whether measurements were taken from distinct samples or whether the same sample was measured repeatedly
☐	☒	The statistical test(s) used AND whether they are one- or two-sided<br>*Only common tests should be described solely by name; describe more complex techniques in the Methods section.*
☒	☐	A description of all covariates tested
☐	☒	A description of any assumptions or corrections, such as tests of normality and adjustment for multiple comparisons
☒	☐	A full description of the statistical parameters including central tendency (e.g. means) or other basic estimates (e.g. regression coefficient) AND variation (e.g. standard deviation) or associated estimates of uncertainty (e.g. confidence intervals)
☐	☒	For null hypothesis testing, the test statistic (e.g. *F*, *t*, *r*) with confidence intervals, effect sizes, degrees of freedom and *P* value noted<br>*Give P values as exact values whenever suitable.*
☒	☐	For Bayesian analysis, information on the choice of priors and Markov chain Monte Carlo settings
☒	☐	For hierarchical and complex designs, identification of the appropriate level for tests and full reporting of outcomes
☒	☐	Estimates of effect sizes (e.g. Cohen's *d*, Pearson's *r*), indicating how they were calculated

Our web collection on statistics for biologists contains articles on many of the points above.

Software and code

Policy information about availability of computer code

Data collection	In vivo imaging data were obtained on a Leica TCS SP5 microscope and acquired through the Leica proprietary software.<br>Lipid droplets data were obtained on a Olympus FV1000 confocal microscope and acquired through the Olympus proprietary software.<br>BODIPY data and images of immuno-labelled brains were obtained on a Nikon A1R confocal and acquired through the Nikon proprietary software.<br>Mitochondria morphology data were obtained on a Leica TCS SP8 STED 3X microscope and acquired through the Leica proprietary software.<br>qRT-PCR data collection and processing was performed using LightCycler 480SW 1.5 (Roche Life Science).
Data analysis	- Statistical analysis was done using GraphPad Prism 8 (GraphPad Software).<br>- ATP in vivo imaging data were analyzed using a custom-written MATLAB script previously published to analyse FRET ratio (Plaçais et al. 2017) using MATLAB R2022a software.<br>- LD and BODIPY analysis were done using Fiji (ImageJ 1.54f) and CellProfiler 4.2.8 Analyst Software.<br>- Mitochondria analysis were done using Icy v2.5.2.0 Software and Fiji (ImageJ 1.54f), a Fiji custom-written macro, previously published in Pavlowsky eta l. 2024, was used to determine the volume of the minimal envelope containing all the mitochondria (Bun 2022, zenodo https://doi.org/10.5281/ZENODO.7118602.)<br>- Figures were designed on Affinity Designer v2.

For manuscripts utilizing custom algorithms or software that are central to the research but not yet described in published literature, software must be made available to editors and reviewers. We strongly encourage code deposition in a community repository (e.g. GitHub). See the Nature Portfolio guidelines for submitting code & software for further information.

Data

Policy information about availability of data

All manuscripts must include a data availability statement. This statement should provide the following information, where applicable:

- Accession codes, unique identifiers, or web links for publicly available datasets
- A description of any restrictions on data availability
- For clinical datasets or third party data, please ensure that the statement adheres to our policy

No data sets that require mandatory deposition into a public database were generated during the current study. Source data that are reported as graphs on figures and extended data figures are available as supplementary Information alongside the paper. Additional raw data, which represent a large volume, will be shared with no restriction by the corresponding author upon request.

Research involving human participants, their data, or biological material

Policy information about studies with human participants or human data. See also policy information about sex, gender (identity/presentation), and sexual orientation and race, ethnicity and racism.

Reporting on sex and gender	not applicable
Reporting on race, ethnicity, or other socially relevant groupings	not applicable
Population characteristics	not applicable
Recruitment	not applicable
Ethics oversight	not applicable

Note that full information on the approval of the study protocol must also be provided in the manuscript.

Field-specific reporting

Please select the one below that is the best fit for your research. If you are not sure, read the appropriate sections before making your selection.

☒ Life sciences ☐ Behavioural & social sciences ☐ Ecological, evolutionary & environmental sciences

For a reference copy of the document with all sections, see nature.com/documents/nr-reporting-summary-flat.pdf

Life sciences study design

All studies must disclose on these points even when the disclosure is negative.

Sample size	Sample size choice was based on previous studies from our group such as Plaçais et al. Nat. Com. 2017 , Silva et al. Nat metabo. 2022, and Pavlowsky et al. 2024 for behavior, live imaging, lipid droplets and mitochondria morphology experiments.
Data exclusions	To avoid giving disproportionate statistical weight to a small number of flies, rare behavioral experiments involving a group of less than 6 flies were excluded
Replication	Each behavioral assay was replicated in at least 2 independent experiments. Imaging experiments were replicated in at least 4 independent experiments. All replications were successful.<br>All quantification were performed unblinded. Statistical parameters including the definitions and exact value of n (e.g. number of group of flies (behavior experiments), number of brains (in vivo live-imaging, LD staining, BODIPY staining and mitochondria morphology), number of experiments replication (RT-qPCR),deviations and p values are reported in the figures and corresponding figure legends. Statistical analysis was carried out using Prims8 (Graph Pad Software). Comparisons between two groups were performed by unpaired two-sided Student's t-test, with results given as the value tx of the t distribution, where x is the number of degrees of freedom. Comparisons among three groups were performed by one-way analysis of variance (ANOVA) with post hoc testing by the Newman-Keuls pairwise comparisons test between the experimental group and its controls (significance: $p<0.05$). ANOVA results are given as the value of the Fisher distribution F(x,y), where x is the number of degrees of freedom numerator and y is the number of degrees of freedom denominator. Data are expressed as the mean ± s.e.m. with dots as individual values corresponding to a group of 40-50 flies analyzed together in a behavioral assay, to the response of a single recorded fly for ATP imaging, to one brain for LD and BODIPY experiments and for mitochondria morphology experiments, and to one mRNA extraction from heads of a group of 50 flies for RT-qPCR experiment.
Randomization	Flies were assigned to experimental groups based on genotypes.
Blinding	Data collection and analysis was not performed blind. Blinding is not applicable because the investigator who set up the experiment is the

Blinding	same person doing the analyses. However, each experiment was associated with proper controls, and sample were collected and analyzed under identical conditions.

Reporting for specific materials, systems and methods

We require information from authors about some types of materials, experimental systems and methods used in many studies. Here, indicate whether each material, system or method listed is relevant to your study. If you are not sure if a list item applies to your research, read the appropriate section before selecting a response.

Materials & experimental systems

n/a	Involved in the study
☐	☒ Antibodies
☒	☐ Eukaryotic cell lines
☒	☐ Palaeontology and archaeology
☐	☒ Animals and other organisms
☒	☐ Clinical data
☒	☐ Dual use research of concern
☒	☐ Plants

Methods

n/a	Involved in the study
☒	☐ ChIP-seq
☒	☐ Flow cytometry
☒	☐ MRI-based neuroimaging

Antibodies

Antibodies used	Primary antibodies:<br>Atto647N FluoTag®-X4 anti-RFP NanoTag cat#N0404-Atto647N-L (dilution: 1/100)<br>rabbit anti-GFP Invitrogen cat# A11122 (dilution: 1/250)<br>nc82 mouse Developmental Studies Hybridoma Bank Cat#nc82 (dilution: 1/100)<br>Secondary antibodies:<br>Alexa Fluor-488 anti-rabbit Invitrogen cat# A11034 (dilution: 1/400)<br>Alexa Fluor-594 anti-mouse Invitrogen cat# A11005 (dilution: 1/400)
Validation	antibody-citations<br>RFP https://doi.org/10.1016/j.cub.2024.03.050<br>GFP https://doi.org/10.1016/j.neuron.2018.03.032<br>nc82 https://dshb.biology.uiowa.edu/nc82

Animals and other research organisms

Policy information about studies involving animals; ARRIVE guidelines recommended for reporting animal research, and Sex and Gender in Research

Laboratory animals	Drosophila melanogaster flies were raised on standard food medium containing yeast, cornmeal and agar, on a 12h:12h light-dark cycle at 18°C with 60% humidity. The Canton-Special (CS) strain was used as the wild-type strain. All lines were out-crossed for at least three generations to flies carrying a CS wild-type background. The study was performed on 0-3-day-old adult flies. For behavior experiments, both male and female flies were used. For imaging experiments, female flies were used.<br>List of Drosophila strains:<br>- UAS-LD-GFP (provided by M. A. Welte)<br>- UAS-AT1.03NL (provided by H. Imamura)<br>- UAS-AT1.03RK25 (provided by H. Imamura)<br>- VT30559-Gal4 (Vienna Drosophila Resource Center VDRC: v206077)<br>- tubulin-GAL80ts; VT30559-Gal4 (Plaçais et al 2017)<br>- elav-Gal4 (Luo et al. 1994)<br>- Repo-Gal4 (Comas et al., 2004)<br>- tubulin-GAL80ts; R54H02-Gal4 (Silva et al. 2022)<br>- UAS-Dicer2 (Bloomington Drosophila Stock Center BDSC: 24650)<br>- Tub-Gal80ts; UAS-Dcr2, VT30559-Gal4 (this study)<br>- UAS-Bmm RNAi GD5139 (VDRC: v37877)<br>- UAS-FABP RNAi KK116001 (VDRC: v109169)<br>- UAS-CPT1 RNAi KK100935 (VDRC: v105400)<br>- UAS-MTPalpha RNAi GD11299 (VDRC: v21845)<br>- UAS-Drp1 GD10456 (VDRC: v44155)<br>- UAS-PDH RNAi KK107865 (VDRC: v104022)<br>- UAS-ACAT1 GD7132 (VDRC: v16099)<br>- UAS-Bmm RNAi JF01946 (BDSC:25926)<br>- UAS-FABP RNAi HMS01163 (BDSC: 34685)<br>- UAS-CPT1 RNAi HMS00040 (BDSC:34066)<br>- UAS-RNAi MTPalpha HMS00660 (BDSC: 32873)<br>- UAS-RNAi HAD1 HMC05280 (BDSC: 62273)

- Had1nl (BDSC: 1037)
- UAS-Drp1 HMC03230 (BDSC: 51483)
- Mi{Trojan-GAL4.1}bmmMI13321-TG4.1 (BDSC:67510)
- UAS-mtDsRed (BDSC: 93056)
- UAS-GLaz RNAi HMC06329 (BDSC: 67228)
- UAS-Tango11 RNAi HMJ30309 (BDSC: 63996)
- UAS-Apolpp RNAi HM05157 (BDSC: 28946)
- UAS-Apoltp RNAi HMC03294 (BDSC: 51937)
- UAS-Lrp1 RNAi HMS02875 (BDSC: 44579)
- UAS-LD-GFP, UAS-Bmm RNAi JF01946 (this study)
- UAS-LD-GFP, UAS-CPT1 RNAi HMS00040 (this study)
- UAS-LD-GFP, UAS-MTPalpha RNAi HMS00660 (this study)
- UAS-AT1.03NL; UAS-MTPalpha RNAi HMS00660 (this study)
- UAS-AT1.03NL, UAS-Drp1 HMC03230 (this study)
- UAS-AT1.03NL, UAS-Drp1 HMC03230; UAS-MTPalpha RNAi HMS00660 (this study)
- UAS-mtDsRed, UAS-Drp1 HMC03230 (this study)
- UAS-CPT1 RNAi KK100935; UAS-Drp1 RNAi GD10456 (this study)
- UAS-PDH RNAi KK107865; UAS-Drp1 RNAi GD10456 (this study)
- UAS-ACAT1 RNAi GD7132; UAS-Drp1 RNAi GD10456 (this study)

Wild animals

The study did not involve wild animal

Reporting on sex

For behavior experiments, mixed populations of males and females were used. For imaging and immuno-labelling experiments, females were used.

Field-collected samples

The study did not involve samples collected from the field

Ethics oversight

No ethical approval or guidance was required since in this study we used Drosophila melanogaster.

Note that full information on the approval of the study protocol must also be provided in the manuscript.

Plants

Seed stocks

Report on the source of all seed stocks or other plant material used. If applicable, state the seed stock centre and catalogue number. If plant specimens were collected from the field, describe the collection location, date and sampling procedures.

Novel plant genotypes

Describe the methods by which all novel plant genotypes were produced. This includes those generated by transgenic approaches, gene editing, chemical/radiation-based mutagenesis and hybridization. For transgenic lines, describe the transformation method, the number of independent lines analyzed and the generation upon which experiments were performed. For gene-edited lines, describe the editor used, the endogenous sequence targeted for editing, the targeting guide RNA sequence (if applicable) and how the editor was applied.

Authentication

Describe any authentication procedures for each seed stock used or novel genotype generated. Describe any experiments used to assess the effect of a mutation and, where applicable, how potential secondary effects (e.g. second site T-DNA insertions, mosiacism, off-target gene editing) were examined.
